# Designing patient-oriented combination therapies for acute myeloid leukemia based on efficacy/toxicity integration and bipartite network modeling

**DOI:** 10.1038/s41389-024-00510-9

**Published:** 2024-03-01

**Authors:** Mehdi Mirzaie, Elham Gholizadeh, Juho J. Miettinen, Filipp Ianevski, Tanja Ruokoranta, Jani Saarela, Mikko Manninen, Susanna Miettinen, Caroline A. Heckman, Mohieddin Jafari

**Affiliations:** 1 Department of Biochemistry and Developmental Biology, University of Helsinki, Helsinki, Finland; 2 Institute for Molecular Medicine Finland (FIMM), HiLIFE, University of Helsinki, Helsinki, Finland; 3 Department of Hematology, Helsinki University Hospital Comprehensive Cancer Center, Helsinki, Finland; 4 Orton Orthopaedic Hospital, Helsinki, Finland; 5 Adult Stem Cell Group, Faculty of Medicine and Health Technology, Tampere University, Tampere, Finland; 6 Tays Research Services, Wellbeing Services County of Pirkanmaa, Tampere University Hospital, Tampere, Finland; 7 Institute for Molecular Medicine Finland - FIMM, HiLIFE - Helsinki Institute of Life Science, iCAN Digital Precision Cancer Medicine Flagship, University of Helsinki, Helsinki, Finland

**Keywords:** Acute myeloid leukaemia, High-throughput screening

## Abstract

Acute myeloid leukemia (AML), a heterogeneous and aggressive blood cancer, does not respond well to single-drug therapy. A combination of drugs is required to effectively treat this disease. Computational models are critical for combination therapy discovery due to the tens of thousands of two-drug combinations, even with approved drugs. While predicting synergistic drugs is the focus of current methods, few consider drug efficacy and potential toxicity, which are crucial for treatment success. To find effective new drug candidates, we constructed a bipartite network using patient-derived tumor samples and drugs. The network is based on drug-response screening and summarizes all treatment response heterogeneity as drug response weights. This bipartite network is then projected onto the drug part, resulting in the drug similarity network. Distinct drug clusters were identified using community detection methods, each targeting different biological processes and pathways as revealed by enrichment and pathway analysis of the drugs’ protein targets. Four drugs with the highest efficacy and lowest toxicity from each cluster were selected and tested for drug sensitivity using cell viability assays on various samples. Results show that ruxolitinib-ulixertinib and sapanisertib-LY3009120 are the most effective combinations with the least toxicity and the best synergistic effect on blast cells. These findings lay the foundation for personalized and successful AML therapies, ultimately leading to the development of drug combinations that can be used alongside standard first-line AML treatment.

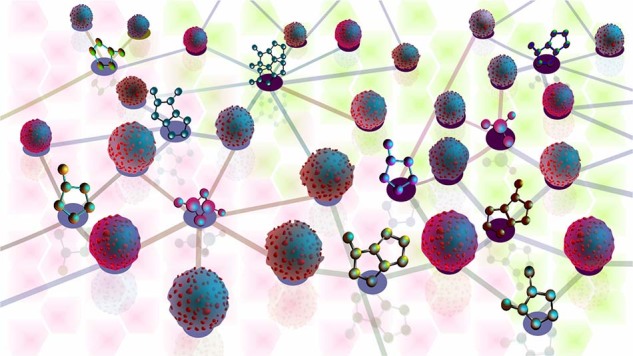

## Introduction

Acute myeloid leukemia (AML) is an inter- and intra-tumor heterogeneous disease [1, 2]. It is identified when the bone marrow (BM) contains at least 20% of blast cells of the myeloid lineage [3]. Traditional chemotherapeutics have limited efficacy in patients over the age of 65, with a survival rate of less than 25% at 1 year follow-up and <9% after 5 years [4]. Despite recent advances in genome sequencing, which enables researchers to identify a large number of mutations, we are still hampered by the absence of drugs that are specifically tailored to target these mutated protein variants in cancer [5]. On the other hand, the majority of AML patients do not have actionable mutations, and the link between cancer genotype, phenotype and therapeutic function of action is poorly understood [6]. Even if we overcome the above difficulties and identify the exact mutations in genotype, monotherapy drug resistance will remain a major clinical complication [7]. Targeted anti-cancer compounds used in combination therapy have the potential to overcome resistance, improve patient response to current treatments, reduce dose-limiting single-agent toxicity, and broaden the spectrum of available therapies by targeting different proteins within pathways [8].

Drug combination therapy offers the chance to suppress a number of pathways synergistically, including patient-specific cancer rescue pathways and phenotypic redundancy across heterogeneous cancer sub-clones [9]. The phenotypic effects of thousands of drug combinations can be evaluated in patient-derived cells and other pre-clinical model systems using high-throughput screening. However, because there are so many possible drug and dose combinations, large-scale multi-dose combinatorial screening is not recommended, due to the limited number of cells available from patient samples. Using the presented method in this study, researchers would be able to categorize the most important AML drugs into different clusters, each of which targets proteins associated with various signaling pathways.

In our earlier research, we designed a systems pharmacology approach based on network modeling to identify prospective drug combinations in AML [10]. To gain a deeper understanding of the factors that govern drug response in AML patients, we utilized a unique and extensive dataset obtained through drug response screening of samples from both AML patients and healthy donors in Finland as part of our study. Our model accounts for the efficacy and toxicity of drug response, which are simultaneously evaluated on patient and healthy samples, respectively [11]. A weighted bipartite network composed of two parts, chemical components, and patient samples was built to develop a drug combination strategy using the screening outcomes of single drug responses on AML patient samples. This enables researchers to directly access the phenotype of the patients’ cancer cells through ex vivo drug response data, and by using network modeling and clustering analysis, demonstrate the drugs’ functionalities. Next, top drug combinations can be predicted based on phenotypic responses of samples in each cluster. In addition, we used two different computational resources, i.e., molecular biology annotations, and the chemical structure of drugs, to perform intra-cluster homogeneity analysis. Subsequent to the design of effective drugs with diverse characteristics for combination therapy, the next step involves the evaluation of the toxicity of combinations.

Considering the importance of toxicity, in this study, we investigated the drug response of AML patient and healthy donor samples to calculate both efficacy and toxicity, respectively. Ex vivo drug response screening was assessed on AML patient and healthy samples using cell viability. Given that the clinical symptoms in patients are caused by blast cell accumulation in bone marrow [12], we suggested the most blast-specific combinations as promising combinations for AML treatment, while having lowest effect on lymphocytes as healthy cells. The comparable efficacy and decreased toxicity observed in the proposed combinations, ruxolitinib-ulixertinib and sapanisertib-LY3009120, prioritize them over first-line combinations in AML, where the majority of blasts are eradicated along with other cell types.

## Materials and methods

Ex vivo drug-response data were generated at the Institute for Molecular Medicine Finland (FIMM) for a prospective series of 252 samples from 186 patients with AML as part of the Functional Precision Medicine Tumor Board-cohort study [11]. The dead cell readouts (CellTox Green, Promega) were extracted from results of experiments that included the drug response of 199 bone marrow samples from AML patients tested against 625 chemical compounds. To determine the inhibition efficacy of each drug on each sample, the mean value of drug response across dosages was extracted after processing. This dataset can be represented as a 199-by-624 matrix, with rows representing samples and columns representing drugs tested on the AML samples. Furthermore, the full submatrix (with no missing entries) of 81 patient samples and 296 chemical compounds was extracted using the NIMMA package [13]. The magnitudes of the dose response levels vary across experimental protocols and techniques due to the heterogeneity of the different platforms on which the high-throughput assays were performed. We normalized the mean value of dose response levels to provide coincident and comparable therapeutic efficacy across different experiments to facilitate downstream use of our dataset. Given that cell death was used in drug sensitivity assays, we calculated the inhibition rate (*R*_*inhibition*_) of cancer cells to drug treatments as a uniform measure using the min–max normalization method:

$${R}_{{inhibition}}=\frac{{celldeath}-\min ({celldeath})}{\max \left({celldeath}\right)-\min ({celldeath})}$$

As a result, one represents the highest sensitivity, and zero represents the lowest sensitivity, since the normalized inhibition rates range from 0 to 1. In matrix *A* each *ij* entry denoted by *a*_*ij*_ indicates the normalized inhibition rate of drug response *j* on sample *i*.

### Reconstruction and analysis of the bipartite network model

A weighted network *G* = (*V, E, ω*) is a triple—a set of three elements—in which *V* is a set of nodes, *E* is a set of edges between nodes in *V*, and *ω* is a function that assigns a weight to each edge $e\in E$. A network is said to be bipartite if *V* can be divided into two sets, *V*_1_, *V*_2_, so that every edge $e\in E$ is connected to a node in *V*_1_ and a node in *V*_2_. A bipartite weighted network is shown as *G* = (*V*_1_*, V*_2_*, E, ω*). Suppose $S=\left\{{s}_{1},{s}_{2},\ldots ,{s}_{m}\right\}$ and $D=\left\{{d}_{1},{d}_{2},\ldots ,{d}_{{mn}}\right\}$ are samples and the drugs sets in the dataset, respectively. The data matrix *A* was used to construct a weighted bipartite network where *V*_1_ = *S* was set of 81 samples, and *V*_2_ = *D* consisted of 296 drugs. The weight of the edge that joins node *s*_*i*_ (sample *i*) and node *d*_*j*_ (drug *j*) was the *ij* entry of the matrix *A*. A weighted bipartite network was built, with two parts: samples and compounds, and weight representing the inhibition rate (*R*_*inhibition*_) as explained above.

### Construction and analysis of the drug similarity network

A bipartite network can be projected into two different types of unipartite networks containing nodes of only one type. The projection of the bipartite network, A, onto the “drug” node set was considered here, and the weight of edge between drug *d*_*i*_ and drug *d*_*j*_ was as follows:

$${w}_{{ij}}=\mathop{\sum }\limits_{k=1}^{81}({a}_{{ik}}\times {a}_{{jk}})$$

This weight was considered as the similarity score between two drugs, *d*_*i*_ and drug *d*_*j*_, according to their efficacy on samples. Only edges with a weight greater than the median of similarities were kept in order to consider them strong enough edges in the projected network. In order to identify functionally similar drugs in terms of drug response the Louvain community detection method [14] was used.

### Computational corroboration

To accomplish intra-cluster homogeneity analysis, we employed two computational methods. The first method identified the significant difference between biological pathways of drug targets’ protein targets at each cluster, while the second evaluated drug chemical structure similarity at each cluster. Using the drug-target common (DTC) database [15], we built a drug-target network, which was a bipartite network in which each link connects drugs to their protein targets.

To better understand the protein targets of drugs in each cluster, we assigned a score to each protein based on the number of distinct drugs targeting that protein in clusters 1 and 2. Let *f*_1*,P*_ (*f*_2*,P*_) denote the number of unique drugs in cluster *C*_1_ (*C*_2_) targeting a particular protein P. The score of protein P, defined by

$$S\left(P\right)=\log \frac{{f}_{1,P}}{{f}_{2,p}}$$

Proteins with a score S greater than log (2) are considered to be preferentially targeted by drugs in cluster 1, denoted by PPT1. Similarly, PPT2 proteins have a score of less than *log*(0.50). The KEGG pathway annotations and biological processes of each cluster’s protein targets were also extracted using clusterprofiler R package [16] and ShinyGO [17]. The KEGG pathway annotations and biological processes provided in the package were used to map pathways and biological processes (GO) to our protein sets PPT1 and PPT2. The settings used in the gseKEGG and gseGO functions were 10,000 permutations, the minimum size of the gene set to test was 10, and the maximum size of the gene set to test was 500. REVIGO was used to summarize the enriched GO terms (http://revigo.irb.hr/). The significantly enriched GO terms (Adj. *P* value < 0.05) were analyzed by REVIGO [18]. This program removes redundant GO terms and the similarity between terms is reflected by semantic space.

A simplified molecular input line entry system (SMILES) of the drug molecules was retrieved to compare the chemical structures of the compounds, and it was then converted into an extended connectivity fingerprint (ECFP) in order to evaluate the dice similarity between the molecules. The dice similarity between molecules A and B is one of the standard metrics for molecular similarity calculations in which

$${S}_{A,B}=2c/(a+b),$$

where a is the number of ON bits in molecule A, b is the number of ON bits in molecule B, and c is the number of ON bits in both A and B molecules [19]. To calculate the dice similarity of the compounds, a simplified molecular input line entry system (SMILES) of the drug molecules was retrieved and transformed into an extended connectivity fingerprint (ECFP). The rcdk package [20] was used to calculate the similarity between chemical compounds [21–23].

We utilised four well-known scoring functions ZIP [24], HSA [21], Bliss [22], and Loewe [23] to assess the potential synergy of drug combinations. The observed drug combination responses in these models were compared with the expected combination responses to quantify synergy of drug combination. The combination ratio (CR) was also defined as the ratio of the response of combinations to the maximum for the two single agents, respectively. By this metric, a CR value of higher than 1 indicates the drug combination is more effective than either single agent [25]. The effect of drug combinations on five dosages (1,10,100,1000,10000 nM) was monitored in this study, and the DECREASE model was used to predict drug combination dose-response at the full matrix. Synergy scores were calculated using the SynergyFinder web application (version 3.0) [26].

### Patient sample processing

Freshly frozen bone marrow mononuclear cells (BM-MNCs) from 16 AML patients were obtained from the Helsinki University Hospital Comprehensive Cancer Center after informed consent (permit numbers 303/13/03/01/2011, Helsinki University Hospital Ethics Committee). Freshly frozen BM-MNCs from healthy donors (*n* = 5) were obtained under approval of the Tampere University Hospital Ethics Committee, Tampere, Finland (R15174). The samples were numbered from 1 to 16 in Supplementary Table S4, from which samples one to five have been used for both CellTiter-Glo (CTG) (Promega) and flow cytometry (FC) analysis. The samples were selected based on clinical blast cell percentage higher than 49%. Following thawing, the cells were cultured in RPMI supplemented with 12.5% HS-5 stromal cell-derived conditioned medium (CM), 10% fetal bovine serum, 2 mM l-glutamine and penicillin/streptomycin and DNAse, then incubated at 37 °C and 5% CO2 for 2-3 hours. After the incubation time the cells were counted and adjusted to a final number of 200,000 cells for each CTG test and 1106 cells/ml for FC analysis. The patient characteristics are presented in Supplementary Table S1.

### Preparation of drug plates

The compounds (Supplementary Table S2) were dissolved in dimethyl sulfoxide (DMSO) and dispensed on 384-well plates (Corning, Corning, NY, USA) using an acoustic liquid handling device Echo 550 (Labcyte, Sunnyvale, CA). DMSO was used as a negative control and 100 µM benzethonium chloride (BzCl) as a positive control (Table S2).

### Cell viability analysis using CTG

The AML cells were seeded on pre-drugged 384-well plates (Corning) containing chemical compounds at five different concentrations in two replicates. The final number of cells in each well was adjusted to 5000 cells in 25 µl per well and incubated for 72 h at 37 °C and 5% CO2. Cell viabilities were assessed using the CTG assay (Promega), and the luminescence signal was measured using a PHERAstar FS plate reader (BMG LABTECH). As quality control, viability screening was used to check how the cells survive in 384-well plates during the 72 h incubation. Viability of the cells was monitored at 0 h and at 72 h using the CTG assay.

### High throughput flow cytometry

For phenotype-based drug sensitivity profiling, a high throughput flow cytometry (HTFC) assay was performed. Following thawing, BMMNCs were seeded using MultiFlow FX RAD (BioTek) to 384-well compound plates (Greiner), 20,000 live cells in 20 µl CM in each well, and incubated for 72 h at 37 °C and 5% CO2 (Figure S1). Monoclonal antibodies for CD45, CD38, CD34, CD117, CD11b, CD14 and CD15, apoptosis dye Annexin-V and dead cell exclusion dye DRAQ7 (Table S7) were added with the Echo 525 acoustic dispenser (Labcyte Inc.) and stained for 30 min at room temperature (Table S3). Cells were analyzed with the iQue3 screener (Sartorius, Germany). ForeCyt software (Sartorius) was used to analyze the remaining viable cells and data normalized to the number of viable cells in the DMSO control wells. Drug sensitivity scores (DSS) and SynergyFinder 3.0 were used to analyze the results [26]. The gating strategy is presented in Supplementary Figure S2.

### Statistical analysis

T-test was used to show that the mean of inter-cluster dice similarities is less than the mean of intra-cluster similarities. We also used a statistical proportion test to show that the proportion of inter-cluster drug combinations with efficacy greater than the third quantile (*Q*_3_ or 75th percentile) of efficacy values and toxicity less than the first quantile (*Q*_1_ or 25th percentile) of toxicity values is significantly higher than the random choices (*probability* = 0.33). This demonstrates that inter-cluster drug combinations have the highest efficacy and the lowest toxicity. A similar approach was utilized for calculating CR values as well as synergy scores. In KEGG, a biological pathway enrichment analysis was calculated based on hypergeometric test followed by false discovery rate (FDR) correction. Fold Enrichment was calculated by dividing the percentage of genes in the list that belong to a pathway by the corresponding percentage in the background. Fold Enrichment indicates how significantly genes from a specific pathway are over-represented [17].

## Results

**Fig. 1:**
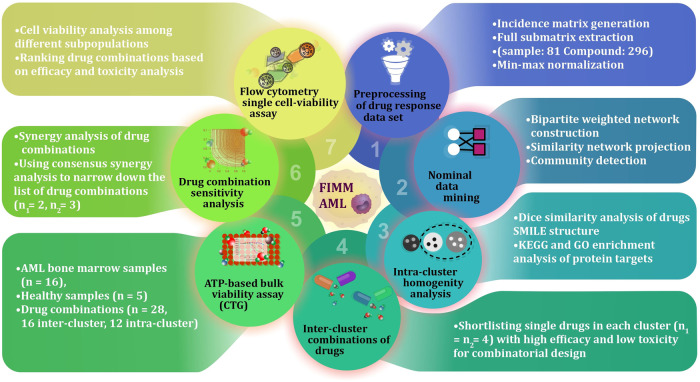
Schematic outline of the study. Data pre-processing began after data collection, which was followed by full matrix extraction, weighted bipartite network reconstruction, and computational validation. After the selection of the best combinations, bone marrow and peripheral blood samples from both healthy individuals (*n* = 5) and AML patients (*n* = 16) were subjected to drug sensitivity assessment. For ATP-based viability assay the study design contains 8 drugs and 28 combinations in 384-well plates, each drug with 5 different concentrations and two replicates. The single cell sensitivity assay using the iQue® Screener PLUS flow cytometer was performed in 384-well plates to monitor drug effects on cell sub-types. The study design contains 5 drugs and 3 combinations, all with two replicates and five concentrations. For sapanisertib, the drug concentrations are 0.1, 1, 10, 100, and 1000 nM, and for all other drugs are 1, 10, 100, 1000, and 10,000 nM.

The entire workflow of this study is depicted in Fig. 1. The drug responses of 625 chemical compounds tested on 199 bone marrow samples from patients with AML were obtained from the FIMM AML data set [11]. The bipartite network was constructed using this data set, as explained in the materials and methods section. A bipartite network can be projected onto two different types of unipartite networks containing nodes of only one type. The projection of the bipartite network, onto the “drug” node set is considered here, called the drug similarity network. The Louvain community detection approach was used to find drugs that behaved similarly in terms of drug response [14]. The results gave us two communities (clusters) of drugs denoted by *C*_1_ and *C*_2_ with network sizes of 155 and 141, respectively (Table S1).

### Comparing AML drug clusters: evaluating protein target pathways and chemical structure similarity

We used two independent computational methods to determine how distinct the two clusters are: the first identifies the significant difference between biological pathways of drug protein targets in each cluster, and the second evaluates the chemical structure similarity of drugs in each cluster. We constructed a drug-target network using the drug target commons (DTC) database [15], which is also a bipartite network in which each link connects drugs to their protein targets. Let *T*_1_ and *T*_2_ represent the set of protein targets of drugs in the cluster *C*_1_ and *C*_2_, respectively, and *T* represents the union of *T*_1_and *T*_2_. In this study |${T}_{1}$|$=921,{|}{T}_{2}|=842,{and|T|}=1055.$

**Fig. 2:**
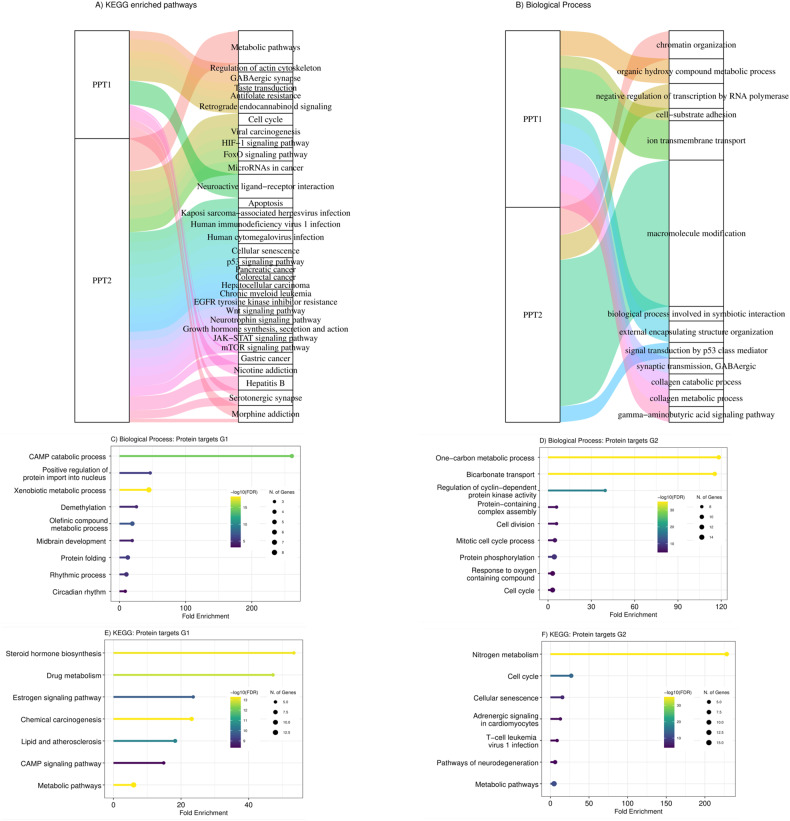
Gene Enrichment Analysis for Proteins in Clusters 1 and 2. Sankey plot of enriched (**A**) KEGG signalling pathways and (**B**) GO biological processes related to target protein clusters PPT1 and PPT2. Each rectangle on the right side represents a pathway or biological process, and the size of each rectangle illustrates the degree of connectivity of each pathway. Each biological process or pathway is represented by a unique color. GO and KEGG pathway enrichment analysis on proteins that are merely targets by drugs in one cluster. G1 (G2) includes proteins that are targeted by at least three drugs in cluster 1 (cluster 2) (155 and 141 drugs). **C** Biological processes (BPs) of G1, (**D**) Biological processes (BPs) of G2, (**E**) KEGG pathway related to G1 proteins, and (**F**) KEGG pathway related to G2 proteins. The size of the node corresponds to number of genes, the x-axis is Fold Enrichment and the color of bars indicates the negative logarithm of Fold Enrichment.

Proteins with a score S (explained in the methods) greater than log (2) are considered to be preferentially targeted by drugs in cluster 1, denoted by PPT1. Similarly, PPT2 proteins have a score of less than $\log (0.50)$. We performed GSEA (gene set enrichment analysis) on PPT1 and PPT2 proteins based on their associated scoring functions. As expected, the biological processes and signaling pathways affected by drugs in Clusters 1 and 2 are distinct. This difference enables us to inhibit two different signaling pathways using one combination. Drugs in cluster 1 (PPT1), such as LY3009120 (a pan-RAF inhibitor), predominantly target proteins associated with the RAF-MEK-ERK signaling pathway. This pathway plays a crucial role in cell proliferation and growth, indirectly influencing processes like cell-substrate adhesion and ion trans-membrane transport, which are enriched in our analysis [27]. In contrast, JAK1/2 inhibitors like ruxolitinib target JAK proteins, involved in cytokine signaling and immune responses, impacting pathways related to neuroactive ligand-receptor interactions and the regulation of actin cytoskeleton [28]. Drugs like birabresib, which target proteins in the bromodomain and extra-terminal (BET) family, have a role in gene regulation through chromatin binding, affecting gene expression and pathways related to chemical reactions and collagen metabolism [29]. Plicamycin, which binds to guanine-cytosine-rich regions of DNA, may influence gene expression and regulation, impacting pathways related to collagen metabolism and other DNA-dependent processes (Fig. 2A) [30]. On the other hand, proteins targeted by drugs in cluster 2 (PPT2) (silmitasertib, ulixertinib, sapanisertib, and teniposide) are in the p53 signaling pathway, cell cycle, apoptosis, and pancreatic, colorectal and chronic myeloid leukemia cancers and related to tumorigenesis and progression pathways, including human immunodeficiency virus 1 infection [31–34].

We also performed ShinyGO [17] Gene Ontology and KEGG pathway enrichment analysis on proteins that are merely targeted by drugs in one cluster. For this purpose, two protein sets G1 and G2 were selected such that G1 includes proteins targeted by at least three drugs in cluster 1 and at most two drugs in cluster 2, and similarly, G2, consist of proteins that are mostly targeted by drugs in cluster 2 (at least three drugs in cluster 2 and at most two drugs in cluster 1). REVIGO was also used to summarize the enriched GO terms, and the results are shown in Fig. 2 and Tables S2 and S3. The cAMP signaling pathway, lipids and atherosclerosis, steroid hormone biosynthesis, and rhythmic processes and circadian rhythm are biological processes related to G1 proteins, which are mostly targeted by LY3009120, birabresib, plicamycin, and ruxolitinib. Cell cycle, cellular senescence, T-cell leukemia virus 1 infection and cell division, mitotic cell cycle, and protein phosphorylation processes are related to G2 proteins, mostly targeted by silmitasertib, ulixertinib, sapanisertib, and teniposide. Therefore, we demonstrate that the protein targets of drugs in each cluster are involved in distinct pathways and biological processes.

**Fig. 3:**
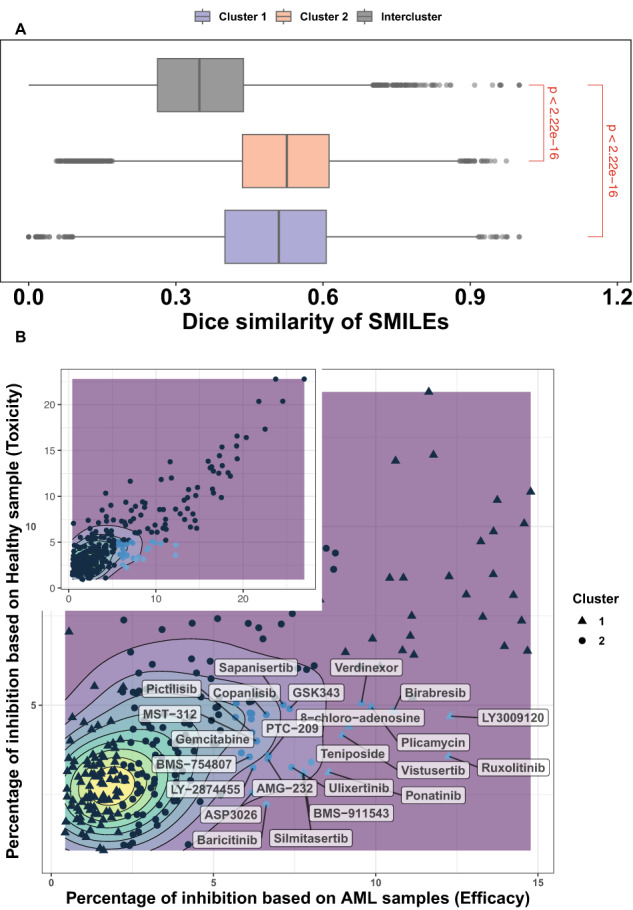
Comparative analysis of dice similarity and drug efficacy-toxicity profiles in AML therapy. **A** Box plot of dice similarity coefficient indices comparing intra-cluster 1 and 2 to inter-cluster compound pairs. P-value is generated using Wilcoxon signed-rank test, shown in red color. **B** The toxicity and efficacy of 296 drugs. Inset plot shows the relationship between toxicity and efficacy. Top five percent of drugs whose toxicity is less than the average of all drug toxicity and whose efficacy is greater than the average of all drug efficacy are in blue, and their name is shown in rectangle labels.

To do homogeneity analysis of chemical structure of drugs, the dice similarity test was used to show how structurally similar the drugs are in each cluster. This measurement compares the number of chemical features shared by a pair of compounds to the average size of the total number of features present. Pairwise similarities were calculated for chemical compounds chosen from two drug clusters for inter-cluster comparison. Drugs from different clusters are less similar than drugs from the same cluster, as shown in Fig. 3A. According to the box plot, the inter-cluster similarities are less than the intra-cluster similarities in both clusters. The results of the *t*-test imply that the mean of inter-cluster similarities is less than the mean of intra-cluster similarities in clusters 1 and 2 (*p*-value < 2.2e-16 for both *t*-test).

### Combination selection: balancing toxicity and efficacy across clusters

As a result, we demonstrated that clusters are well-separated and that the protein targets of drugs in each cluster are involved in distinct pathways. In this novel combination strategy, we aim to select two drugs from distinct clusters while taking both toxicity and efficacy into account. The optimal combinations are those that have lower toxicity than the average toxicity and higher efficacy values than the average efficacy value for all drugs. For each drug, the average drug response of healthy and AML patient samples in the data set are considered as toxicity and efficacy, respectively. We assume that the ideal drugs have no inhibitory effect on healthy samples but significantly influence blast cells in AML patient samples. We chose the top 5% of drugs whose toxicity is less than the average of all drug toxicity and efficacy is greater than the average of all drug efficacy. Figure 3B depicts the link between toxicity and efficacy values of 296 drugs on 81 samples. The top four selected small molecules in each cluster are summarized in Table 1 and Table S4. Four chemical compounds from cluster 1 including birabresib, LY3009120, plicamycin, and ruxolitinib as well as four drugs from cluster 2 including sapanisertib, silmitasertib, teniposide, and ulixertinib were chosen for drug combination testing. According to our experimental design, the combination of drugs within a single cluster is known as negative group or intra-cluster, and the combination of drugs between clusters is considered as positive group or inter-cluster.

**Table 1:** The selected chemical compounds from two clusters of drugs in the drug similarity network.

Cluster 1	Cluster 2
LY3009120 (LY30)	Teniposide (Teni)
Ruxolitinib (Ruxo)	Silmitasertib (Silm)
Birabresib (Bira)	Ulixertinib (Ulix)
Plicamycin (Plic)	Sapanisertib (Sapa)

### Enhanced efficacy and reduced toxicity in inter-cluster drug combinations on AML patient samples revealed by cell viability drug screening

In the testing of all 16 inter-cluster and 12 intra-cluster combinations at five different concentrations, the cell viability of 16 samples from AML patients and 5 samples from healthy donors were monitored. Patient samples with blast percentage more than 49% were chosen for testing with the CTG assay (Table S5). The average inhibition across dosages on 16 patient samples is regarded as efficacy, whereas the average inhibition across dosages on healthy samples is regarded as toxicity. The drug combinations with rectangular labels have higher efficacy and lower toxicity than the median. The proportion test (p-value = 0.006) revealed that the percentages of inter-cluster drug combinations with high efficacy (efficacy higher than the third quantile of efficacy values) and low toxicity (toxicities lower than the first quantile of toxicities) are significantly more than random choices.

**Fig. 4:**
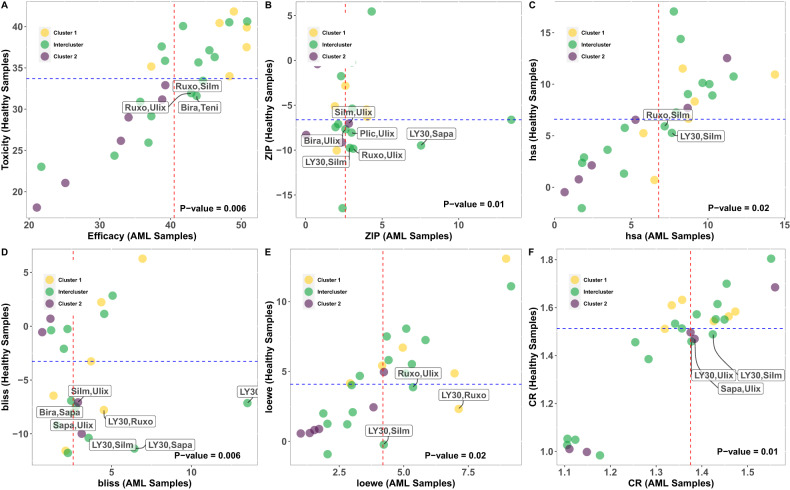
Drug combinations’ synergy scores on 16 AML samples and 5 healthy samples. The *X*-axis depicts the synergy in AML samples and the *Y*-axis represents the synergy in healthy samples. The median inhibition on AML and healthy samples is shown by dashed lines in red and blue, respectively. There are three groupings: clusters 1, 2, and intercluster, and the color of each dot indicates each of these groups. The *p* values presented in each panel are associated with the proportion test, comparing the inter-cluster combination with the random selection of drugs. The average of inhibition of drug combinations on dosages (**A**) and several synergy scores were depicted in separate panels using synergy scoring functions ZIP (**B**), HSA (**C**), Bliss (**D**), Loewe (**E**), and combination ratio (CR) of drug combinations on AML and healthy samples (**F**).

The synergy and combination ratio (CR) of drug combinations on AML and healthy samples was then calculated using synergy scoring functions HSA [21], Bliss [22], Loewe [23], and ZIP [24] (Figs. 4 and S3). The same analysis was done on synergy scoring values, and it was discovered that inter-cluster drug combinations differ significantly from random choices (P-values shown in Fig. 4A-F). The drug combinations shown with rectangular labels have the highest synergy on AML patient samples, and the lowest synergy on healthy samples. Table 2 summarizes all six plots and the significant drug combinations according to different measures are highlighted by green (inter-cluster), yellow and purple (intra-clusters). Following CTG analysis, consensus across synergy scoring functions led to the selection of the five best drug combinations out of 28 to quantify blast-specific drug responses with flow cytometry. Additionally, we used one of the most extensive databases, the Probes & medications portal (PDP) dataset [35], to extract the protein targets of these selected drugs. Table S6 provides a summary of the hypergeometric test findings, which show that there is no discernible overlap between the protein targets of these drugs whether taken separately or in combination. The need for future work arises to assess relevant biomarkers of on-target activity for each single and combination approach.

**Table 2:** Selected drug combinations sorted by synergy scoring functions.

Highlighted in green (inter-cluster), yellow (intra-cluster 1), and purple (intra-cluster 2). Inh stands for the average of inhibition of drug combinations on dosages, ZIP, hsa, bliss, and loewe are synergy scorings and CR is the combination ratio.

### Cell subtype viability analysis highlights low toxicity of selected combinations

Using the CTG assay, we measure the general BM-MNC sensitivity, whereas with flow cytometry analysis we measure the number of live cells among different cell populations. Following 72-hour treatment with the 5 selected combinations on 3 different samples, viability of different cell subtypes of interest was measured by flow cytometry. Sample selection was based on the inclusion of three biological replicates for each combination, considering available cell numbers to enhance statistical power and result reliability. For each sample, there is a specific plate layout which can be found in Supplementary Fig. S1. We used six cell surface markers (CD14, CD15, CD45, CD38, CD117, and CD34; Table S7) to identify the major leukocyte populations present in the AML BM-MNCs: monoblasts, myelocytes, leukemic blasts, leukemic stem cells, and myeloid progenitor cells (Fig. S2).

**Fig. 5:**
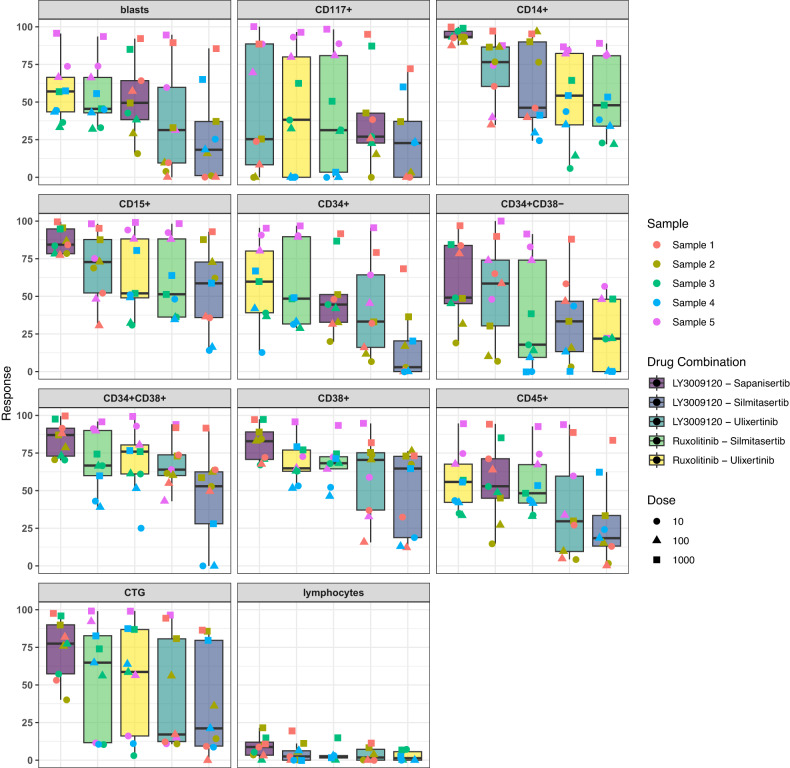
The cell viability assay (CTG) and response of different cell populations to 5 selected combinations using flow cytometry assay. Response signifies the percentage of dead cells following a 72 h treatment. The number of cells in each well was counted and normalized by the min–max normalization method. For each combination, three different samples, distinguished by the color of points, were treated with three different doses (10, 100, and 1000 nM), which are illustrated by the different point shapes. The colors in each cell group facet corresponds to a specific drug combination.

**Fig. 6:**
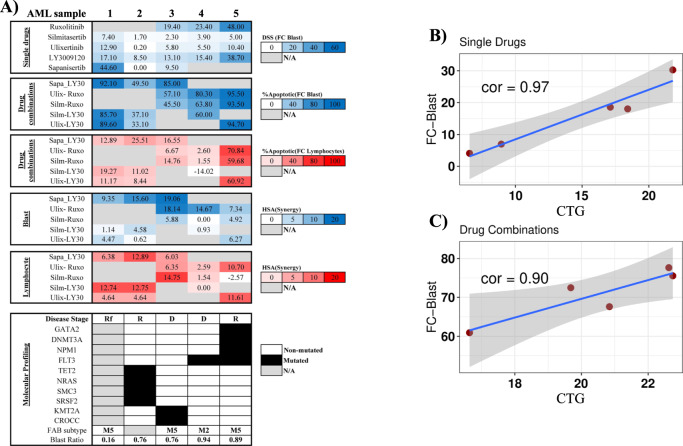
Characteristics of drug responses and correlation analysis in AML treatment: Comprehensive flow cytometry and CTG assessment. **A** A heat map showing characteristics of single agent and combination responses measured by flow cytometry readout. Blast-specific response of single drugs is highlighted according to drug sensitivity score (DSS) values with dark blue corresponding to high DSS value and white to low DSS value. Blast-specific and lymphocyte-specific response combinations at 1000 nM are highlighted according to percentage of apoptotic/dead cells, with dark blue in blast and red for lymphocyte corresponding to high percentage and white to a low percentage of apoptotic/dead cells. The synergistic effect of the drug combination was assessed based on the HSA synergistic score in 1000 nM on blast cells shown in blue and lymphocytes shown in red. **B** The correlation between responses measured by CTG and flow cytometry on five single drugs ruxolitinib, silmitasertib, ulixertinib, LY3009120, and sapanisertib, and (**C**) five drug combinations sapanisertib-LY3009120, ulixertinib-ruxolitinib, silmitasertib-ruxolitinib, silmitasertib-LY3009120, and ulixertinib-LY300912.

In the studied samples, the average of blasts out of CD45 positive leukocytes, was 70% in DMSO, while on average 36% ± 16% of the blasts were killed by the combinations (Table S8). Based on the results, the percentage of dead cells for all five combinations in lymphocytes is considerably lower than 25% (Fig. 5). More importantly selected combinations have lower synergistic effect on lymphocytes compared to the blast population, demonstrating the lower toxicity of combinations (Figs. 6 and S3). The combination of JAK1/2 inhibitor (ruxolitinib) with either ERK or CSNK2A1 inhibitor had the highest efficacy and lowest toxicity, demonstrating the important role of these targets in AML. Numerous studies show the significance of the JAK/STAT signaling system in determining how hematopoietic cells react to various cytokines and growth factors [36, 37]. Recently there has been increased interest in different drug combinations with ruxolitinib [38–41] and as our results show the combinations of this drug, by having the lowest toxicity, seem to be promising for AML treatment.

### Blast-specific drug responses in AML: Efficacy profiles of selected combinations

**Fig. 7:**
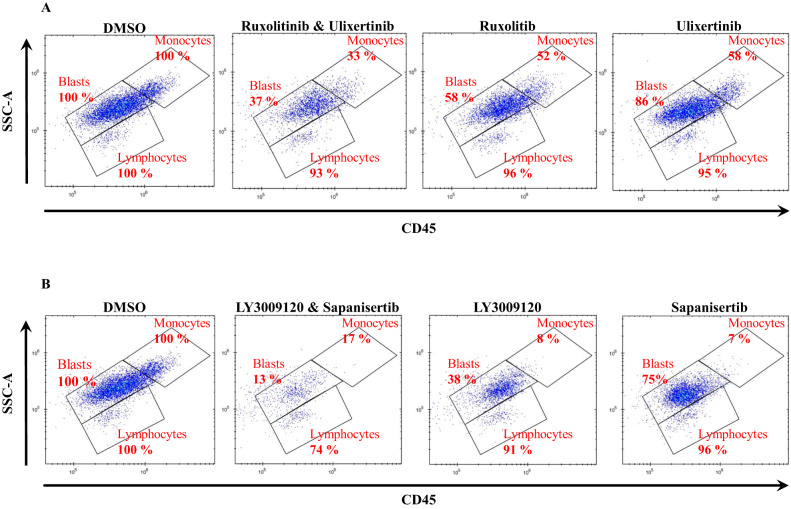
Flow cytometry scatter plots showing the effects of drug combinations on cell populations, along with comparisons to DMSO and single drug treated samples. **A** This figure illustrates the effects of ruxolitinib and ulixertinib combination and (**B**) LY3009120 and sapanisertib combination on blasts, monocytic cells (CD14+) and lymphocytes after 72 h drug treatment. Numbers represent the percentage of cell counts in each population in comparison with untreated control. The plot represents a concentration of 1000 nM on sample AML_3.

We were able to assess blast-specific drug combination responses and compare them to the other combinations within different samples. Among the five tested combinations, two combinations with ruxolitinib which targets JAK1/2 were among the most efficient combinations. The combination of ruxolitinib with ulixertinib, an ERK inhibitor, exhibits the strongest efficacy against blasts, according to the results. After treatment, the combination induced 47% ± 13% cell death in blasts (Fig. 5 and Table S8) with a more synergistic effect on the blast population compared to the lymphocyte population (Fig. 6A). We depicted the gating of 1000 nM concentration of each drug on sample AML_3 to better understand the impact of combination therapy vs. DMSO control and single drug treated samples in Fig. 7. The number of blast cells in the ruxolitinib and ulixertinib treated well was reduced to 37%, showing the largest reduction compared to all other treatments, as shown in Fig. 7A. The second combination of ruxolitinib and silmitasertib, a CSNK2A1 inhibitor, showed high efficacy on blasts. On average, this combination induced death to almost half ± 14% of the blast population but had less effect on lymphocytes (Fig. 5 and Table S8). Additionally, this combination had a substantially higher inhibition rate compared to each single drug and acted synergistically toward the blast population (Fig. 6A).

Given the importance of pan-RAF inhibition, we next examined LY3009120 in combination with three other drugs. The samples used for the combination of LY3009120 and sapanisertib (mTOR1/2 inhibitor), consist of 56% blast and the response for them is 40% ± 12% inhibition. To confirm that this combination is efficient, we analyzed the effect of LY3009120 and sapanisertib combination with single treated and DMSO-treated cells in AML_3. In the combination-treated sample, the blast cells were significantly reduced to 13% while in the individual drugs LY3009120 and sapanisertib reduced the blasts to 38% and 75%, respectively (Fig. 6B). These results indicate that this combination has substantially higher inhibition rate compared to each single drug and a greater synergistic effect on blasts than on lymphocytes (Fig. 6A). Ulixertinib (ERK inhibitor) is the second drug that was used in combination with pan-RAF inhibitor. Patient samples treated with this combination, on average, contained 60% blast cells and after treatment they are reduced to 28% ± 14%. Finally, we tested the combination of LY3009120 with silmitasertib, a CSNK2A1 inhibitor on three different samples. The average blast population for these three samples is 62% and the response was 21% ± 5%. Overall, as shown in Figs. 5 and 6A, all combinations have very little impact on the lymphocyte populations, demonstrating low toxicity, and significantly more impact on less differentiated malignant cells, demonstrating the efficacy of the combinations.

### Increased sensitivity of AML samples to combination therapies over single drugs, regardless of genetic mutations and prognosis categories

There is a significant correlation between CTG assay and blast specific results, indicating that reduction in cell number measured by CTG, is related to the malignant cell populations (Figs. 6B and 6C). The cell viability readout for a single drug is converted to a drug sensitivity score (DSS) which is a drug sensitivity metric based on area under the dose-response curve. A greater DSS indicates higher sensitivity [42]. Strikingly, by combining selected inter-cluster drugs, the blasts were targeted, and combinations showed a synergistic effect on this population (Fig. 6A). Considering the most prevalent mutations among AML patients [43, 44], we examined the existing mutations in selected samples to monitor the drug responses based on genetic changes (Fig. 6A). To evaluate the impact of the combinations on samples bearing genetic alterations, some mutations that are frequently found in AML patients were considered (Fig. 6A). Mutation to *FLT3*, a well-known driver gene in AML was represented in two samples. Other prevalent mutations occurred in *NPM1, GATA2, DNMT3A, TET2, KMT2A, NRAS, SMC3*, and *SRSF2*. The combinations induced a synergistic effect on the blast population, regardless of the genetic alterations. The European Leukemia Network (ELN) classifies patients into three prognosis categories: “favorable”, “intermediate”, or “adverse” [45]. AML patients are also classified using the French-American-British (FAB) classification [46], which is based on morphological features. Regardless of sample type, we observed a synergistic effect following treatment. Importantly, after therapy, we noticed a synergistic effect in all samples, indicating that these combinations are effective at combating the heterogeneity of AML. It has been demonstrated that drugs should target the less differentiated leukemic blasts to achieve the best response in patients [6]. Given these two observations—the presence of the most relevant mutations and the prevalence of blast cells in the samples— the combinations seem to be promising for treatment.

### Efficacy and toxicity of the novel combinations compared to first-line treatment in AML

**Fig. 8:**
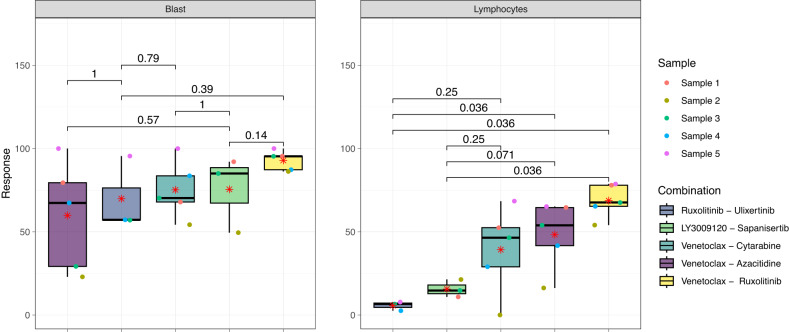
Flow cytometry assay of selected combinations compared to first-line AML combinations. Response signifies the percentage of dead cells following 72 h treatment. The count of cells in each well was adjusted relative to the count in control wells featuring both positive (DMSO) and negative (BzCl) controls using the min–max normalization method. Each combination has been tested on different samples at 50 nM concentration for venetoclax and 1000 nM for the other drugs. Red asterisks define the average response for each combination and colored dots represent different samples. Each panel also represents six p-values resulting from the Wilcoxon signed-rank tests to compare statistically two proposed combinations with three other combinations (including two first-line treatments and one investigational combination) for AML.

In the following analysis, we compared the proposed combinations in this study (ruxolitinib-ulixertinib and LY3009120-sapanisertib) with two FDA-approved combinations for AML (venetoclax-azacitidine and venetoclax-cytarabine), as well as the investigational combination of venetoclax-ruxolitinib. As illustrated in Fig. 8, venetoclax-ruxolitinib demonstrates the highest efficacy on both blast cells and lymphocytes compared to the other combinations. This dual efficacy profile is a noteworthy advantage; however, it comes at the cost of heightened toxicity, as indicated by our results.

Conversely, the novel combinations, ruxolitinib-ulixertinib and LY3009120-sapanisertib, showed comparable efficacy in targeting blast populations as the established combinations. Notably, there was no significant difference in terms of efficacy (p-values are shown in Fig. 8). However, these two combinations have a significant advantage in demonstrating lower toxicity compared to first-line combinations, particularly for lymphocytes. The effects of ruxolitinib-ulixertinib and LY3009120-sapanisertib on blast lymphocyte population were significantly lower than all other combinations except for venetoclax-cytarabine (*p*-value = 0.25) which is not significant but still lower. This reduction in toxicity suggests these combinations can offer effective treatment while minimizing side effects associated with current therapies.

## Discussion

In this study, we employed a nominal data mining approach to construct a weighted bipartite network for the selection of the most effective, as well as the least toxic drugs. We analyzed a substantial dataset consisting of 625 chemicals and 252 patient samples from a large AML-cohort project in Finland [11] spanning the years 2011 to 2019 (the final size of the matrix after preprocessing is 296×81). Importantly, following evaluation on well-annotated samples, we tested the combinations of ruxolitinib-ulixertinib and sapanisertib-LY3009120 and found that these are equivalently effective but less toxic in comparison to established therapies on samples.

Understanding the reaction of both healthy and cancer cells to drugs is a multifaceted subject, encompassing many different variables crucial for assessing the effectiveness and safety of single drugs and drug combinations [47]. The inherent variability in the response of healthy and cancer cells to combination treatment adds a layer of complexity, potentially impacting overall efficacy [48]. The challenges in drug response within AML patients arise from the impact of patient genotype heterogeneity, and germline variation, along with common factors such as age and sex, leading to certain subpopulations exhibiting resistance to identified combination therapies. Microenvironmental influences on cell response, coupled with potential complex drug interactions, contribute to variations in toxicity and efficacy [49]. Additionally, comparisons between single-agent and combination therapies are inherently complex due to dose equivalency [50, 51]. These limitations draw attention to the complex processes that underlies drug responses in cancer and underscore the necessity of using complex techniques in treatment efforts.

To address concerns related to response variations and genetic heterogeneity, our study includes experimental data, providing a comprehensive view of a drug candidate’s performance. The selection method was driven by criteria developed from studies on drug synergy, which allowed us to rank combinations according to cumulative evaluation of efficiency and toxicity [50, 52]. As illustrated in Fig. S3, it is evident that even at lower concentrations, like higher dosages, a synergistic effect is observed. This implies that fine-tuning the dosage could preserve efficacy while potentially mitigating toxicity on normal cells. By cell population-specific drug response shown in Fig. 5 we introduce two combinations having high efficacy on the blast population (malignant cells) and low efficacy on lymphocytes (healthy cells). However, variations in drug response between different samples remain. Due to different factors influencing drug response in patients with AML such as age, genetic variation, and mutations, employing this approach still presents limitations in addressing this challenge. It is imperative to acknowledge that addressing the limitations of this study requires additional and more profound analyses at both the dose level and with more patient-specific focus.

The selected combinations inhibit important signaling pathways and include drugs targeting pan-RAF, JAK1/2, Bromodomain and Extra-Terminal (BET) motif protein family, topoisomerase II, CSNK2A1, ERK, mTOR1/2, and DNA binding. The MAPK (RAS/RAF/MEK/ERK) signaling pathway is hyperactivated in AML patients, leading to leukemogenesis, leukemia progression, and chemo resistance [53–56]. Targeting RAS and ERK poses challenges, making pan-RAF inhibitors a novel and intriguing pharmacological class [57, 58]. Recent studies demonstrated that the pan-RAF inhibitor LY3009120 induces growth inhibition and apoptosis in RAS-mutated AML cell lines [59, 60]. The ERK inhibitor ulixertinib shows early efficacy in treating tumors with MAPK pathway alterations, prevalent in 30% of all human cancers due to activating mutations in RAS, BRAF, or MAP2K1 (MEK1) [61, 62]. LY3214996 proves effective in delaying or reversing resistance to BRAF and MEK inhibitors, and a synergistic effect was observed when combined with pan-RAF inhibitor LY3001920 in a KRAS-mutant colorectal cancer model [63]. Moreover, here we identified ulixertinib and LY3009120 as an inter-cluster combination with high efficacy and low toxicity. Upon further analysis, we found two combinations of ulixertinib and three combinations of LY3009120 among the five most efficient and synergistic combinations.

JAK signaling plays critical roles in several intracellular signaling pathways, and is implicated in leukemias with described aberrations in the JAK/STAT pathway and constitutive STAT activation [64–66]. Ruxolitinib, a non-selective JAK1/2 inhibitor approved for the treatment of myelofibrosis, reduces JAK-signal transducer activation and lowers STAT transcription signaling [67, 68]. Based on computational analysis, CTG-based viability results, and flow cytometry analysis, we revealed that the combinations of ruxolitinib with ulixertinib and silmitasertib are the most blast-specific compounds, while having minor effect on lymphocytes in AML by ex vivo screening.

Lastly, we thoroughly compared the two FDA-approved combinations (venetoclax-azacitidine and venetoclax-cytarabine) and one investigational combination (venetoclax-ruxolitinib) with the novel combinations suggested in this study, namely ruxolitinib-ulixertinib and LY3009120-sapanisertib. Notably, the outcomes validated the comparability of the suggested combinations’ efficacy with first-line combinations in this investigation. There is no significant difference in efficacy on blast cells, between proposed combinations and first-line AML combinations. Significantly, the toxicity of selected combinations is lower than others, except for venetoclax-cytarabine, which indicates that proposed combinations might offer both effective treatment and a reduced side effect compared to standard AML combinations.

In summary, we proposed effective drug combinations for AML patients with the highest efficacy and lowest toxicity based on nominal data mining method and ex vivo drug sensitivity assay. Our results indicate that ruxolitinib-ulixertinib and sapanisertib-LY3009120 could be effective combinations for AML, having the highest synergistic effect, the highest efficacy on blasts, and the lowest toxicity. Although the approach of combining targeted agents suffers from cumulative toxicity effects [69], we demonstrated that our approach overcomes this limitation in designing a drug combination in AML. Nevertheless, our choice of drug candidates for combination therapy prioritized minimizing toxicity; but this serves as a starting point to explore the acceptable toxicity levels associated with various combination approaches among different patient profiles. Considering the importance of toxicity, in all steps we regarded toxicity as an important factor for the selection of combinations with the lowest effect on healthy cells. Standard chemotherapy kills most of the blasts as well as other cell types and has a high value of toxicity [6], while recommended combinations in this study are effective on blasts, but have lower toxicity on other cell populations.

### Supplementary information

supplementary file

## Data Availability

The data that support the findings of this study and all code for data analysis are openly available at https://github.com/jafarilab/DrugComb_AML.

## References

[1] Bowman RL, Busque L, Levine RL (2018). Clonal hematopoiesis and evolution to hematopoietic malignancies. Cell Stem Cell.

[2] Grzywa TM, Paskal W, Wodarski PK (2017). Intratumor and intertumor heterogeneity in melanoma. Transl Oncol.

[3] Bennett JM, Catovsky D, Daniel M-T, Flandrin G, Galton DAG, Gralnick HR (1976). Proposals for the classification of the acute leukaemias French-American-British (FAB) co-operative group. Br J Haematol.

[4] Peipert JD, Efficace F, Pierson R, Loefgren C, Cella D, He J (2022). Patient-reported outcomes predict overall survival in older patients with acute myeloid leukemia. J Geriatr Oncol.

[5] Daver N, Wei AH, Pollyea DA, Fathi AT, Vyas P, DiNardo CD (2020). New directions for emerging therapies in acute myeloid leukemia: the next chapter. Blood Cancer J.

[6] Kuusanmki H, Leppä AM, Pölönen P, Kontro M, Dufva O, Deb D (2020). Phenotype-based drug screening reveals association between venetoclax response and differentiation stage in acute myeloid leukemia. Haematologica.

[7] Intlekofer AM, Shih AH, Wang B, Nazir A, Rustenburg AS, Albanese SK (2018). Acquired resistance to IDH inhibition through trans or cis dimer-interface mutations. Nature.

[8] Lopez JS, Banerji U (2017). Combine and conquer: challenges for targeted therapy combinations in early phase trials. Nat Rev Clin Oncol.

[9] Ianevski A, Lahtela J, Javarappa KK, Sergeev P, Ghimire BR, Gautam P (2021). Patient-tailored design for selective co-inhibition of leukemic cell subpopulations. Sci Adv.

[10] Jafari M, Mirzaie M, Bao J, Barneh F, Zheng S, Eriksson J (2022). Bipartite network models to design combination therapies in acute myeloid leukaemia. Nat Commun.

[11] Malani D, Kumar A, Brck O, Kontro M, Yadav B, Hellesy M (2022). Implementing a functional precision medicine tumor board for acute myeloid leukemiaaml functional molecular precision medicine. Cancer Discov.

[12] Sarma A, Hazarika M, Das D, Kumar Rai A, Sharma JD, Bhuyan C (2015). Expression of aberrant CD markers in acute leukemia: A study of 100 cases with immunophenotyping by multiparameter flowcytometry. Cancer Biomark.

[13] Jafari M, Chen C, Mirzaie M, Tang J. NIMAA: an R/CRAN package to accomplish NomInal data Mining AnAlysis. bioRxiv 10.1101/2022.01.13.475835 (2022).

[14] Blondel VD, Guillaume J-L, Lambiotte R, Lefebvre E (2008). Fast unfolding of communities in large networks. J Stat Mech: Theory Exp.

[15] Tang J, Ravikumar B, Alam Z, Rebane A, Vh-Koskela M, Peddinti G (2018). Drug target commons: a community effort to build a consensus knowledge base for drug-target interactions. Cell Chem Biol.

[16] Wu T, Hu E, Xu S, Chen M, Guo P, Dai Z (2021). clusterProfiler 4.0: a universal enrichment tool for interpreting omics data. Innovation.

[17] Ge SX, Jung D, Yao R (2020). ShinyGO: a graphical gene-set enrichment tool for animals and plants. Bioinformatics.

[18] Supek F, Bošnjak M, Škunca N, Šmuc T (2011). REVIGO summarizes and visualizes long lists of gene ontology terms. PLoS ONE.

[19] Bajusz D, Rcz A, Hberger K (2015). Why is Tanimoto index an appropriate choice for fingerprint-based similarity calculations?. J Cheminform.

[20] Guha R (2007). Chemical informatics functionality in R. J Stat Softw.

[21] Berenbaum MC (1989). What is synergy?. Pharmacol Rev.

[22] Bliss CI (1939). The toxicity of poisons applied jointly 1. Ann Appl Biol.

[23] Loewe S (1953). The problem of synergism and antagonism of combined drugs. Arzneimittelforschung.

[24] Yadav B, Wennerberg K, Aittokallio T, Tang J (2015). Searching for drug synergy in complex dose-response landscapes using an interaction potency model. Comput Struct Biotechnol J.

[25] Kurtz SE, Eide CA, Kaempf A, Khanna V, Savage SL, Rofelty A (2017). Molecularly targeted drug combinations demonstrate selective effectiveness for myeloid-and lymphoid-derived hematologic malignancies. Proc Natl Acad Sci USA.

[26] Ianevski A, Giri, AK, Aittokallio, T. SynergyFinder 3.0: an interactive analysis and consensus interpretation of multi-drug synergies across multiple samples. Nucleic Acids Res. 2022;50:W739–W743. 10.1093/nar/gkac382 PMC9252834 35580060

[27] Park J, Park H, Byun JM, Hong J, Shin D-Y, Koh Y (2021). Pan‑RAF inhibitor LY3009120 is highly synergistic with low‑dose cytarabine, but not azacitidine, in acute myeloid leukemia with RAS mutations. Oncol Lett.

[28] Klein K, Stoiber D, Sexl V, Witalisz-Siepracka A (2021). Untwining anti-tumor and immunosuppressive effects of JAK inhibitors—a strategy for hematological malignancies?. Cancers.

[29] Gilan O, Rioja I, Knezevic K, Bell MJ, Yeung MM, Harker NR (2020). Selective targeting of BD1 and BD2 of the BET proteins in cancer and immunoinflammation. Science.

[30] Albertini V, Jain A, Vignati S, Napoli S, Rinaldi A, Kwee I (2006). Novel GC-rich DNA-binding compound produced by a genetically engineered mutant of the mithramycin producer Streptomyces argillaceus exhibits improved transcriptional repressor activity: implications for cancer therapy. Nucleic Acids Res.

[31] Blanco-Aparicio C, Carnero A (2013). Pim kinases in cancer: diagnostic, prognostic and treatment opportunities. Biochem Pharmacol.

[32] Hayes TK, Der CJ. Targeting the Raf-MEK-ERK mitogen-activated protein kinase cascade for the treatment of RAS mutant cancers. *Ras Superfamily Small G Proteins: Biology and Mechanisms 1: General Features, Signaling*, 135-56 (2014).

[33] Shimizu T, Kuboki Y, Lin C-C, Yonemori K, Yanai T, Faller DV, et al. A phase 1 study of Sapanisertib (TAK-228) in East Asian patients with advanced nonhematological malignancies. Targeted Oncol. 2022;1–10. 10.1007/s11523-021-00855-w PMC8994735 34843044

[34] Economides MP, McCue D, Borthakur G, Pemmaraju N (2019). Topoisomerase II inhibitors in AML: past, present, and future. Expert Opin Pharmacother.

[35] Skuta C, Popr M, Muller T, Jindrich J, Kahle M, Sedlak D (2017). Probes & drugs portal: an interactive, open data resource for chemical biology. Nat Methods.

[36] Ferrajoli A, Faderl S, Ravandi F, Estrov Z (2006). The JAK-STAT pathway: a therapeutic target in hematological malignancies. Curr Cancer Drug Targets.

[37] Eghtedar A, Verstovsek S, Estrov Z, Burger J, Cortes J, Bivins C (2012). Phase 2 study of the JAK kinase inhibitor ruxolitinib in patients with refractory leukemias, including postmyeloproliferative neoplasm acute myeloid leukemia. Blood J Am Soc Hematol.

[38] Borate U, Saultz JN, Kaempf A, Minnier J, Tognon CE, Kurtz SE (2021). Novel combination therapy of venetoclax and ruxolitinib in the treatment of patients with relapsed/refractory acute myeloid leukemia. Blood.

[39] Verbeke D, Gielen O, Jacobs K, Boeckx N, De Keersmaecker K, Maertens J, et al. Ruxolitinib synergizes with dexamethasone for the treatment of T-cell acute lymphoblastic leukemia. Hemasphere 2019;3:e310. 10.1097/HS9.0000000000000310. 10.1097/HS9.0000000000000310 PMC6924552 31976483

[40] Dal Bello R, Pasanisi J, Joudinaud R, Duchmann M, Pardieu B, Ayaka P (2022). A multiparametric niche-like drug screening platform in acute myeloid leukemia. Blood Cancer J.

[41] Karjalainen R, Pemovska T, Popa M, Liu M, Javarappa KK, Majumder MM (2017). JAK1/2 and BCL2 inhibitors synergize to counteract bone marrow stromal cell-induced protection of AML. Blood J Am Soc Hematol.

[42] Yadav B, Pemovska T, Szwajda A, Kulesskiy E, Kontro M, Karjalainen R, et al. Quantitative scoring of differential drug sensitivity for individually optimized anticancer therapies. Sci Rep. 2014;4:5193. 10.1038/srep05193 PMC4046135 24898935

[43] Papaemmanuil E, Gerstung M, Bullinger L, Gaidzik VI, Paschka P, Roberts ND (2016). Genomic classification and prognosis in acute myeloid leukemia. N. Engl J Med.

[44] Tyner JW, Tognon CE, Bottomly D, Wilmot B, Kurtz SE, Savage SL (2018). Functional genomic landscape of acute myeloid leukaemia. Nature.

[45] Rausch C, Rothenberg-Thurley M, Dufour AM, Schneider S, Gittinger H, Sauerland MC (2022). Validation of the 2022 European Leukemianet genetic risk stratification of acute myeloid leukemia. Blood.

[46] Hasserjian RP (2021). Controversies in the recent (2016) World Health Organization classification of acute myeloid leukemia. Best Pract Res Clin Haematol.

[47] Medeiros BC (2018). Interpretation of clinical endpoints in trials of acute myeloid leukemia. Leuk Res.

[48] Dry JR, Yang M, Saez-Rodriguez J (2016). Looking beyond the cancer cell for effective drug combinations. Genome Med.

[49] Mansoori B, Mohammadi A, Davudian S, Shirjang S, Baradaran B (2017). The different mechanisms of cancer drug resistance: a brief review. Adv Pharm Bull.

[50] Meyer CT, Wooten DJ, Paudel BB, Bauer J, Hardeman KN, Westover D (2019). Quantifying drug combination synergy along potency and efficacy axes. Cell Syst.

[51] Xia X (2020). Drug efficacy and toxicity prediction: an innovative application of transcriptomic data. Cell Biol Toxicol.

[52] Ling A, Huang RS (2020). Computationally predicting clinical drug combination efficacy with cancer cell line screens and independent drug action. Nat Commun.

[53] Ricciardi MR, McQueen T, Chism D, Milella M, Estey E, Kaldjian E (2005). Quantitative single cell determination of ERK phosphorylation and regulation in relapsed and refractory primary acute myeloid leukemia Tandem-duplicated Flt3 constitutively activates STAT5 and MAP kinase and introduces autonomous cell growth in IL-3-dependent cell lines. Leukemia.

[54] Steelman LS, Franklin RA, Abrams SL, Chappell W, Kempf CR, Bsecke J (2011). Roles of the Ras/Raf/MEK/ERK pathway in leukemia therapy. Leukemia.

[55] Chang, F, Steelman, LS, Lee, JT, Shelton, JG, Navolanic, PM, Blalock, WL et al. Signal transduction mediated by the Ras/Raf/MEK/ERK pathway from cytokine receptors to transcription factors: potential targeting for therapeutic intervention. Leukemia. 2003;17:1263–93. 10.1038/sj.leu.2402945 12835716

[56] Kornblau SM, Womble M, Qiu YH, Jackson CE, Chen W, Konopleva M (2006). Simultaneous activation of multiple signal transduction pathways confers poor prognosis in acute myelogenous leukemia. Blood.

[57] Cox AD, Fesik SW, Kimmelman AC, Luo J, Der CJ (2014). Drugging the undruggable RAS: Mission possible?. Nat Rev Drug Discov.

[58] Tambe M, Karjalainen E, Vh-Koskela M, Bulanova D, Gjertsen BT, Kontro M (2020). Pan-RAF inhibition induces apoptosis in acute myeloid leukemia cells and synergizes with BCL2 inhibition. Leukemia.

[59] Khoury JD, Tashakori M, Yang H, Loghavi S, Wang Y, Wang J (2020). Pan-RAF inhibition shows anti-leukemic activity in RAS-mutant acute myeloid leukemia cells and potentiates the effect of sorafenib in cells with FLT3 mutation. Cancers.

[60] Park J, Park H, Byun JM, Hong J, Shin D-Y, Koh Y (2021). Pan-RAF inhibitor LY3009120 is highly synergistic with low-dose cytarabine, but not azacitidine, in acute myeloid leukemia with RAS mutations. Oncol Lett.

[61] FDA Updates Highlighting the Latest Cancer Treatments. Oncology Times. 2019;41:36–7. 10.1097/01.COT.0000557874.12684.64.

[62] Roberts PJ, Der CJ (2007). Targeting the Raf-MEK-ERK mitogen-activated protein kinase cascade for the treatment of cancer. Oncogene.

[63] Bhagwat SV, McMillen WT, Cai S, Zhao B, Whitesell M, Shen W (2020). ERK inhibitor LY3214996 targets ERK pathway-driven cancers: a therapeutic approach toward precision medicine. Mol Cancer Ther.

[64] Ihle JN, Kerr IM (1995). Jaks and Stats in signaling by the cytokine receptor superfamily. Trends Genet.

[65] Rawlings JS, Rosler KM, Harrison DA (2004). The JAK/STAT signaling pathway. J Cell Sci.

[66] Lin TS, Mahajan S, Frank DA (2000). STAT signaling in the pathogenesis and treatment of leukemias. Oncogene.

[67] Furqan M, Mukhi N, Lee B, Liu D (2013). Dysregulation of JAK-STAT pathway in hematological malignancies and JAK inhibitors for clinical application. Biomark Res.

[68] McKeage K (2015). Ruxolitinib: a review in polycythaemia vera. Drugs.

[69] Tannock IF, Hickman JA (2019). Molecular screening to select therapy for advanced cancer?. Ann Oncol.

