# Supplementary material for: Designing patient-oriented combination therapies for acute myeloid leukemia based on efficacy/toxicity integration and bipartite network modeling
Source: Oncogenesis. 2024 Mar 1;13(1):11. doi: 10.1038/s41389-024-00510-9 (PMC10907624; doi:10.1038/s41389-024-00510-9)
Supplement: Supplementary file 1 — supplementary file [file 41389_2024_510_MOESM1_ESM.docx]

**Designing patient-oriented combination therapies for acute myeloid leukemia based on efficacy/toxicity integration and bipartite network modeling**

**Mehdi Mirzaie^a,†^, Elham Gholizadeh^a,†^, Juho J. Miettinen^b^, Filipp Ianevski^b^, Tanja Ruokoranta^b,c^, Jani Saarela^b^, Mikko Manninen^d^, Susanna Miettinen^e,f^, Caroline A. Heckman^g,*^, and Mohieddin Jafari^a,*^**

aDepartment of Biochemistry and Developmental Biology, University of Helsinki, Helsinki, Finland.

bInstitute for Molecular Medicine Finland (FIMM), HiLIFE, University of Helsinki, Helsinki, Finland.

cDepartment of Hematology, Helsinki University Hospital Comprehensive Cancer Center, Helsinki, Finland.

dOrton Orthopaedic Hospital, Helsinki, Finland.

eAdult Stem Cell Group, Faculty of Medicine and Health Technology, Tampere University, Tampere, Finland.

fTays Research Services, Wellbeing Services County of Pirkanmaa, Tampere University Hospital, Tampere, Finland.

gInstitute for Molecular Medicine Finland - FIMM, HiLIFE - Helsinki Institute of Life Science, iCAN Digital Precision Cancer Medicine Flagship, University of Helsinki, Helsinki, Finland.

Figures

[Figure 1. Plate layout for FC assay on patient samples. 4](#_Toc148548516)

[Figure 2. Gating strategy of cell populations. 5](#_Toc148548517)

[Figure 3. Synergy analysis across dosage levels. 6](#_Toc148548518)

Tables

[Table 1. List of drugs in each cluster 6](#_Toc154591279)

[Table 2. Biological processes 7](#_Toc154591280)

[Table 3. KEGG pathways 8](#_Toc154591281)

[Table 4. Compound list 9](#_Toc154591282)

[Table 5. Patient characteristics 10](#_Toc154591283)

[Table 6. Protein target analysis of five investigated drugs 10](#_Toc154591284)

[Table 7. Antibodies used in flow cytometry 10](#_Toc154591285)

[Table 8. FC results in different populations 11](#_Toc154591286)


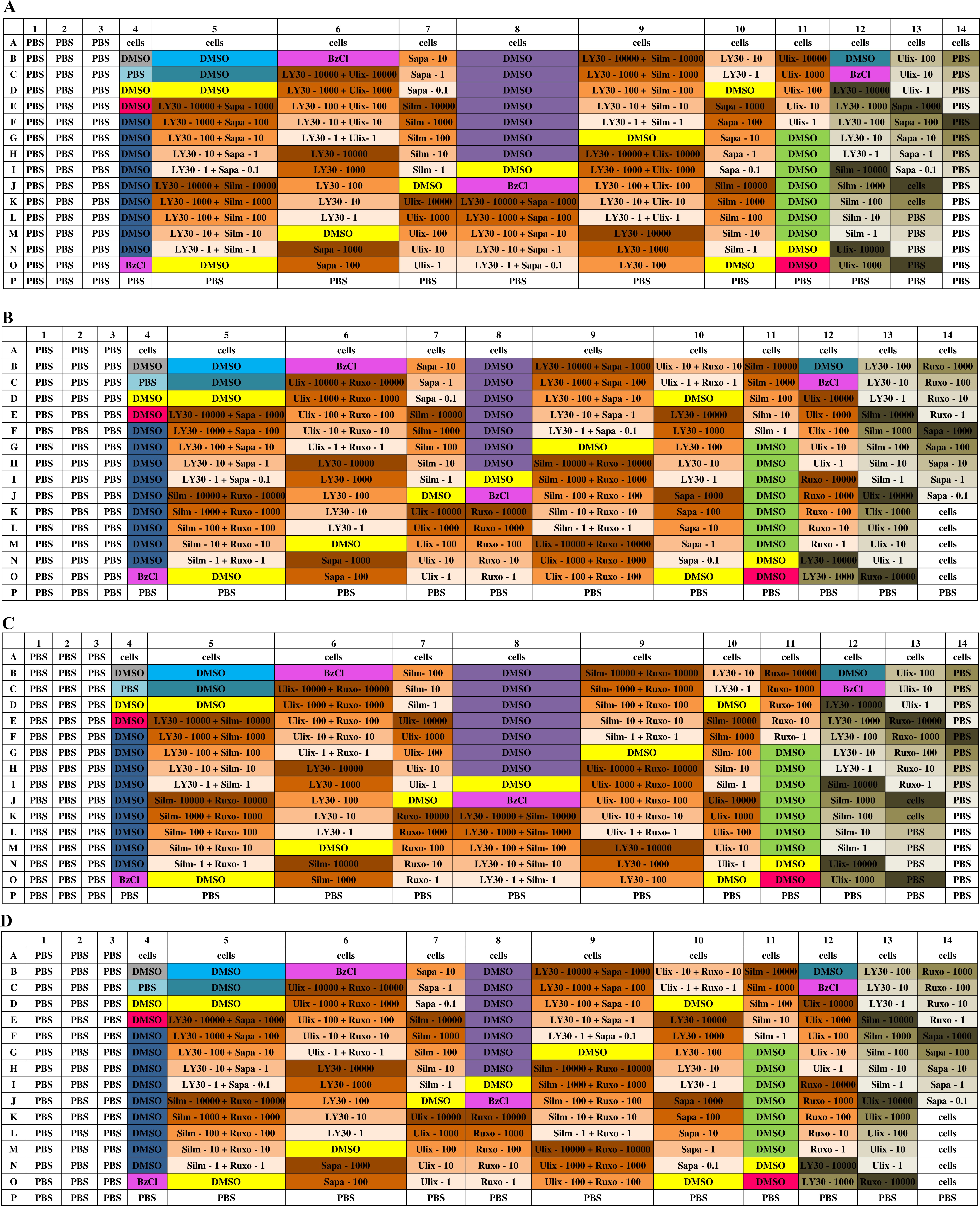


**Figure 1. Plate layout for FC assay on patient samples.**

This figure represents the 384-well plate drug design for sample (A)AML_1 and AML_2, (B)AML_3, (C) AML_4 and (D) AML_5. These layouts contain two replicates for each compound with 5 different concentrations, sapanisertib: 0.1, 1, 10, 100, and 1000 nM, and all other drugs: 1, 10, 100, 1000, and 10000 nM. LY3009120 (LY30), Teniposide (Teni), Ruxolitinib (Ruxo), Silmitasertib (Silm), Birabresib (Bira), Ulixertinib (Ulix), Plicamycin (Plic), Sapanisertib (Sapa).


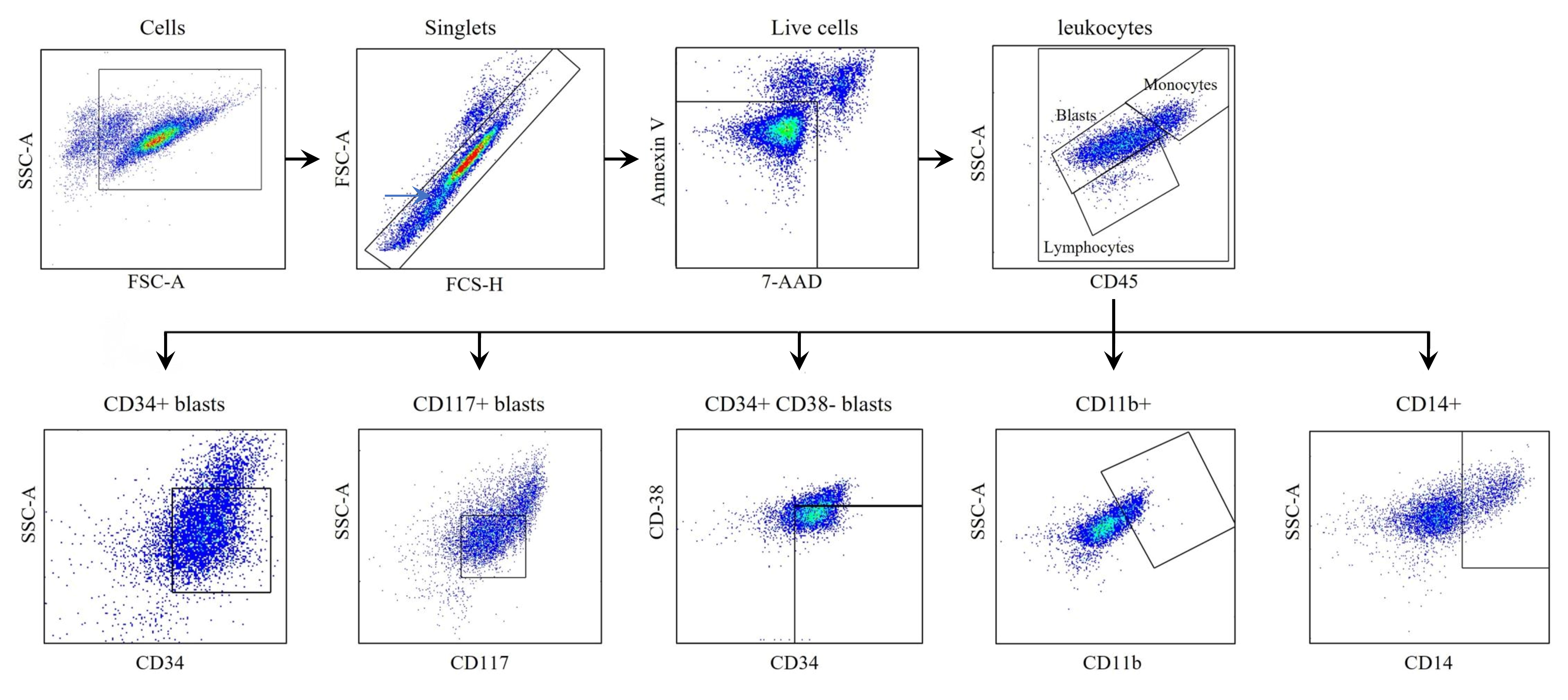


**Figure 2. Gating strategy of cell populations.**

Cells were gated and debris removed based on SSC-A/FSC-A after singlets were identified with FSC-A/FSC-H. Viable cells were gated by excluding the apoptotic and dead cells using Annexin V and DRAQ7, respectively. SSC-A/CD45 was used to gain an overview from the cell composition of the AML sample: blasts – SSC^low^/CD45^dim^, lymphocytes – SSC^low^/CD45^bright^, and monocytic cells – SSC^mid^/CD45^bright^. Blasts were identified with CD34 and CD117 antibodies and leukemic stem cells were gated as CD34+/CD38-. Additional markers CD14 and CD11 were used to gate cells differentiated towards monocytic and granulocytic lineages.


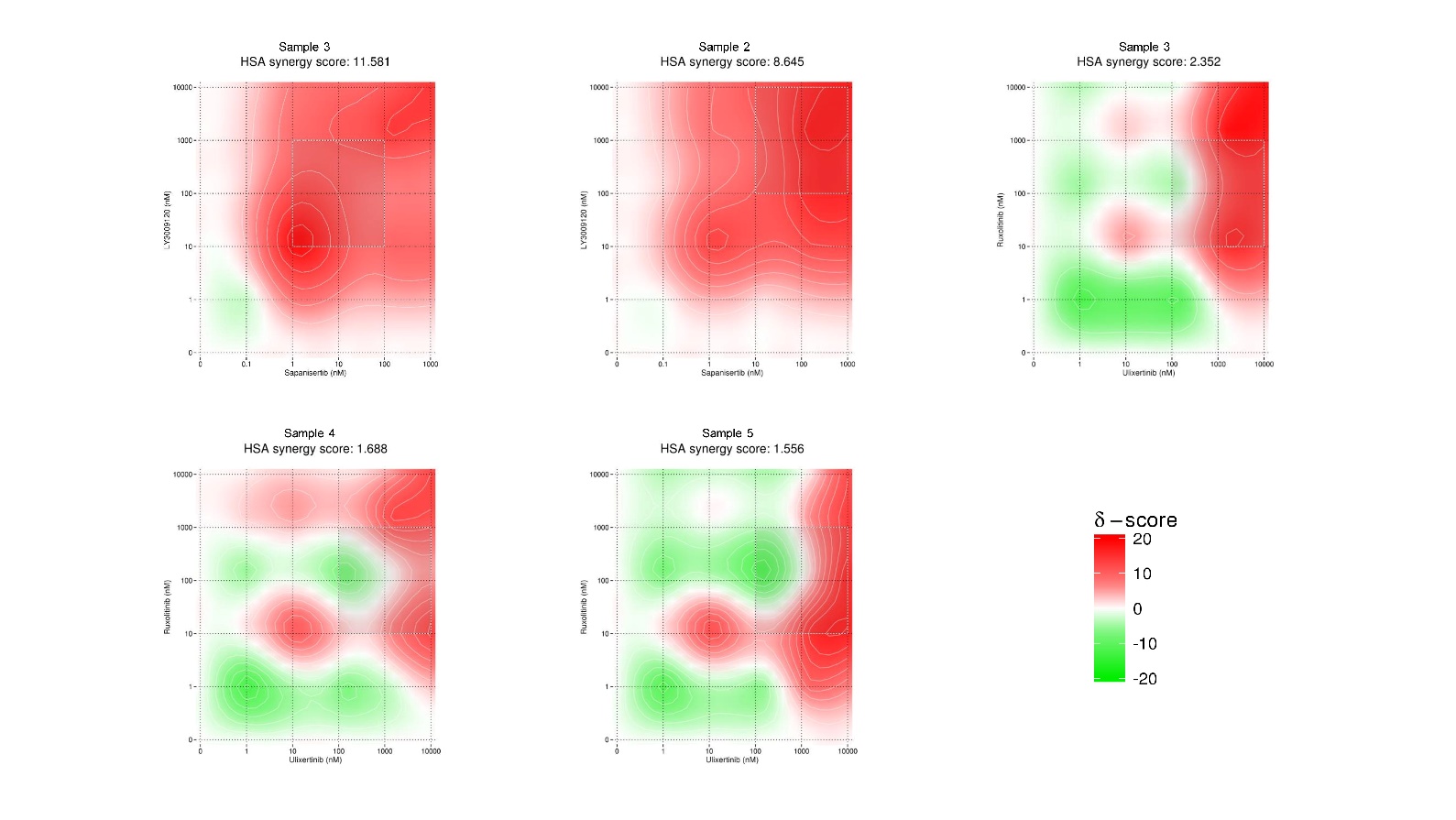


**Figure 3. Synergy analysis across dosage levels.**

This figure presents the synergy analysis results across a range of dosage levels for the reported drug combinations in AML blast populations. Each combination's synergy values are depicted at various dosage points, offering a comprehensive view of the impact of dosage on synergistic responses. The dashed line square represents the highest synergistic region suggested by synergy finder software. The y-axis and x-axis illustrate the dosage levels for each drug, and synergy is depicted as color. Red color represents higher synergy and green represents lower synergy level.

**Table 1. List of drugs in each cluster**

| Cluster 1 | SGI-1776; Apatinib; Cediranib; Dabrafenib; Encorafenib; Icotinib; Lovastatin; Neflamapimod; Talmapimod; Tandutinib; Pravastatin; 1-methyl-D-tryptophan; Brivanib; Linifanib; Perifosine; Dovitinib; Fludarabine; Quisinostat; Valrubicin; MK-8745; VE-821; 8-chloro-adenosine; PF-4800567; AZD-8186; Alectinib; Capmatinib; Golvatinib; Sapitinib; TAK-285; Tesevatinib; Tucatinib; Vandetanib; BMS-777607; Roxadustat; EPZ-5687; GSK-J4; GSK2801; Lomeguatrib; Mocetinostat; Sepantronium bromide; Barasertib; Ibrutinib; Pemetrexed; AZ191; Tazemetostat; AZD4547; Ruboxistaurin; Infigratinib; Methotrexate; Birabresib; CEP-37440; GSK2656157; Marimastat; Verdinexor; Vismodegib; Tamoxifen; GDC-0919; Orteronel; Tasquinimod; BGB324; Galiellalactone; VER 155008; Everolimus; Vorinostat; Floxuridine; Pentostatin; Temozolomide; Allopurinol; Pomalidomide; Goserelin; Letrozole; Thalidomide; Enzastaurin; Lapatinib; Panobinostat; Valproic acid; ENMD-2076; Megestrol acetate; Rabusertib; Metformin; Plerixafor; Pazopanib; Losmapimod; Epacadostat; GSK2879552; Tacrolimus; Pracinostat; Cisplatin; Flutamide; Oxaliplatin; PH-797804; Rocilinostat; URB597; KU-60019; PHA 408; UCN-01; Vatalanib; AZD7545; GNE-7915; Tideglusib; Omipalisib; Tepotinib; Plicamycin; Tretinoin; Oprozomib; Vistusertib; Mubritinib; NMS-873; Pinometostat; Entinostat; Mitotane; Alvocidib; CUDC-305; BIIB021; Onalespib; BMS-911543; Chloroquine; Toremifene; LY3009120; Fingolimod; Doxorubicin; Aldoxorubicin; Ganetespib; IOX-2; Spebrutinib; TEW-7197; Bortezomib; Triciribine; Resminostat; Cladribine; Bimatoprost; Momelotinib; Ponatinib; Cilengitide; NVP-BGT226; Crenolanib; Ruxolitinib; PF-670462; UNC2881; SAR405838; Omacetaxine; Dexamethasone; Givinostat; PF-06463922; PCI-34051; GSK923295; CPI-613; Anastrozole; AR-42; Cerdulatinib; 8-amino-adenosine; AZD-5438; Afuresertib; Bosutinib; Enzalutamide |
| --- | --- |
| Cluster 2 | Hydroxyfasudil; Cobimetinib; Trametinib; AZD-5363; Bentamapimod; GSK-690693; RAF-265; TAK-901; Tivantinib; Ralimetinib; Regorafenib; Nilotinib; Erlotinib; Imatinib; Midostaurin; Vemurafenib; Crizotinib; Doramapimod; Masitinib; Seliciclib; Palbociclib; Neratinib; Pictilisib; Teniposide; SCH772984; PS-1145; Alpelisib; GSK-1070916; Lucitanib; Palomid-529; PF-00562271; Telatinib; Quizartinib; Sunitinib; GDC-0623; C646; GSK343; RGFP966; SGC-CBP30; SGC0946; Tubacin; Tubastatin A; Amuvatinib; Tamatinib; AZD1480; Cabozantinib; Fostamatinib; PF-04708671; Arsenic(III) oxide; Mirdametinib; AVN944; TH588; AT-406; PAC-1; Taselisib; FRAX486; Molibresib; AMG-232; PTC-209; UM729; MST-312; Glasdegib; GSK2830371; Varespladib; NVP-LGK974; Temsirolimus; Paclitaxel; Cytarabine; Rucaparib; Ixabepilone; Thioguanine; Azacitidine; Mitomycin; Methylprednisolone; Lenalidomide; Sonolisib; Triapine; Rociletinib; Fulvestrant; Ulixertinib; Ridaforolimus; Sirolimus; TGX-221; Axitinib; Carboplatin; WEHI-539; Foretinib; Dasatinib; Indibulin; GSK650394; Atorvastatin; Sapanisertib; APR-246; Gemcitabine; Saracatinib; AZ 3146; ML323; Itraconazole; LY-2874455; StemRegenin 1; Gedatolisib; Bafetinib; Celecoxib; Motesanib; Birinapant; Buparlisib; Filanesib; Volasertib; GSK269962; BI 2536; MK-2206; Abemaciclib; Copanlisib; Binimetinib; BMS-754807; Raloxifene; Nilutamide; Bleomycin; Simvastatin; Gefitinib; Baricitinib; Tosedostat; Tarenflurbil; Olaparib; TRAM-34; Sotrastaurin; Ipatasertib; Aminoglutethimide; GSK-2334470; Tacedinaline; Sonidegib; AZD7762; Silmitasertib; CEP-32496; Hydroxyurea; ASP3026; Veliparib; Docetaxel; Linsitinib; Niraparib; Uprosertib |

**Table 2. Biological processes**

| **Description** | **protein_group** | **weight** | **protein_list** |
| --- | --- | --- | --- |
| chromatin organization | PPT2 | 25 | Q9Y6K1, Q9UMN6, Q9H5I1, Q8WTS6, Q8NEZ4, Q86X55, Q53H47, Q09028, Q6ZN18, Q15022, Q92800, Q16576, P26358, O43463, O14744, O14686, O00167, Q6PL18, Q53GL7, P06400, Q9NR48, Q92831, Q09472, O95696, Q8IXJ6 |
| organic hydroxy compound metabolic process | PPT1 | 22 | P42330, Q06520, P50225, P49888, P23141, P0DMM9, O43704, Q9UBM7, P14550, P13569, Q9NYA1, Q9NRA0, P31213, P18405, P19099, P15538, P05107, P14324, Q9NYB5, P17405, P11413, P00374 |
| negative regulation of transcription by RNA polymerase II | PPT2 | 22 | P84022, P10828, P24864, P24385, Q96T88, P40763, Q9Y6K1, Q9H5I1, Q6ZN18, Q15022, O75530, Q92800, P26358, O43463, Q92793, P09874, O75604, P06400, Q09472, P21675, Q9Y5X4, Q8IXJ6 |
| cell-substrate adhesion | PPT1 | 11 | P18084, P08514, P18564, P05556, P08648, P07237, P20701, P05107, P17301, P50281, P39900 |
| ion transmembrane transport | PPT1 | 35 | P05556, P03905, O43497, P13569, Q9NRA0, O15440, Q7RTT9, Q9Y694, Q96NT5, Q8TCC7, Q4U2R8, P41440, P15328, P14207, P11413, Q96SW2, P46098, P43681, O75762, P47869, Q16445, P18505, P18507, P47870, O00591, P34903, Q8N1C3, P78334, Q99928, P31644, O14764, P48169, P14867, P28472, Q9UN88 |
| macromolecule modification | PPT2 | 130 | P53350, Q8TD08, Q13627, P29317, Q9HCP0, Q8TDC3, Q5VT25, Q9P1W9, Q14012, O43293, Q7KZI7, Q13131, O14757, Q9BZL6, P31749, O14965, Q9UEE5, Q9H2X6, Q96SB4, Q8IW41, O75582, Q9HAZ1, Q13188, P51955, P51817, O96017, O96013, P27448, Q13153, P23443, Q9Y243, Q16513, P31751, Q96GD4, P17612, Q05655, P51946, O75116, P04637, Q8NEB9, P22612, Q8WWL7, O95067, P68400, O96020, P25440, P49768, P06748, P10415, Q8TEK3, O00141, P51812, O15530, P67870, P36507, P11274, P05771, P20248, Q02750, P14635, Q15078, P24864, P24385, Q13526, P78396, Q13563, Q8IV63, Q9H2K2, P78527, Q96T88, P40225, Q13490, Q15118, Q96B36, Q9Y6K1, Q9UMN6, Q9H5I1, Q99873, Q8WTS6, Q8NEZ4, Q86X55, Q53H47, Q09028, Q15022, O75530, Q92800, Q16576, P26358, O60678, O43463, O14744, O14686, P13500, P31947, O00571, O00167, Q92793, P18031, P09874, Q9H237, Q9Y6F1, Q9UGN5, Q9NR21, O95271, Q9H0J9, Q8N3A8, Q7Z3E1, Q53GL7, Q2NL67, P42574, O75604, P40189, P08887, P06400, Q9NR48, Q92831, Q09472, P21675, O95696, Q9HC16, O60725, Q53EL6, Q9NTG7, Q8IXJ6, P05067, Q6UB28, P50579, Q9UIQ6, P53582, P55786 |
| biological process involved in symbiotic interaction | PPT1 | 13 | P11142, P08238, P18084, P18564, P05556, P08648, P23284, P07237, P05362, P17301, P17405, O75351, P61073 |
| external encapsulating structure organization | PPT1 | 19 | P05556, P08253, P07477, Q9Y5R2, Q9UNA0, Q9NRE1, Q9NPA2, P51512, P51511, P50281, P45452, P39900, P22894, P09238, P09237, O75173, O60882, O14672, P27487 |
| signal transduction by p53 class mediator | PPT2 | 14 | O14757, P31749, O14965, Q9H2X6, Q8IW41, O96017, Q96GD4, P04637, P10415, O00141, O14744, O75604, Q09472, P21675 |
| synaptic transmission, GABAergic | PPT1 | 14 | P35348, P34972, P28222, P47869, Q16445, P18507, P47870, P34903, Q8N1C3, P78334, Q99928, P31644, P48169, P14867 |
| collagen catabolic process | PPT1 | 14 | P05556, P08253, Q9Y5R2, Q9NRE1, Q9NPA2, P51512, P51511, P50281, P45452, P39900, P22894, P09238, P09237, O60882 |
| collagen metabolic process | PPT1 | 15 | P05556, P08253, P17301, Q9Y5R2, Q9NRE1, Q9NPA2, P51512, P51511, P50281, P45452, P39900, P22894, P09238, P09237, O60882 |
| gamma-aminobutyric acid signaling pathway | PPT1 | 14 | P08908, P47869, Q16445, P18505, P18507, P47870, P34903, Q8N1C3, P78334, Q99928, P31644, P48169, P14867, P28472 |

**Table 3. KEGG pathways**

| **Description** | **protein_group** | **weight** | **protein_list** |
| --- | --- | --- | --- |
| Metabolic pathways | PPT2 | 47 | O00764, Q9Y2D0, Q9ULX7, Q8N1Q1, Q16790, P43166, P35218, P34913, P23280, P22748, P09917, P07451, P00915, P00403, O43570, O14684, P32320, P27707, P04183, O00142, P29474, Q9Y6K1, Q9H5I1, Q8WTS6, Q8NEZ4, Q53H47, Q92800, P26358, O43463, O14686, P23921, P36537, P19224, P48426, Q9NR48, Q9NTG7, Q8IXJ6, P30519, P28838, P15144, Q9UNK4, Q9NZK7, Q9NZ20, Q9BZM2, Q9BZM1, P14555, O15496 |
| Regulation of actin cytoskeleton | PPT1 | 12 | P18084, P08514, P18564, P05556, P08648, P20701, P05107, P17301, P61073, P62136, P36873, P61160 |
| GABAergic synapse | PPT1 | 17 | P46459, P47869, Q16445, P18505, P18507, P47870, O00591, P34903, Q8N1C3, P78334, Q99928, P31644, O14764, P48169, P14867, P28472, Q9UN88 |
| Taste transduction | PPT1 | 11 | P46098, P28566, P28222, P28221, P08908, P47869, Q16445, P34903, P31644, P48169, P14867 |
| Antifolate resistance | PPT1 | 11 | O15438, O15440, P31939, Q96NT5, Q05932, P41440, P22102, P15328, P14207, P04818, P00374 |
| Retrograde endocannabinoid signaling | PPT1 | 20 | P03905, O00519, Q9Y4D2, Q99685, P47869, Q16445, P18505, P18507, P47870, O00591, P34903, Q8N1C3, P78334, Q99928, P31644, O14764, P48169, P14867, P28472, Q9UN88 |
| Cell cycle | PPT2 | 17 | O96017, P51946, P04637, Q8WWL7, O95067, O96020, P20248, P84022, P14635, P24864, P24385, P78396, P78527, P31947, Q92793, P06400, Q09472 |
| Viral carcinogenesis | PPT2 | 18 | P17612, P04637, P22612, O96020, P20248, P62993, P24864, P24385, P78396, P40763, O00571, Q92793, P42574, Q9Y6K9, P40189, P06400, Q92831, Q09472 |
| HIF-1 signaling pathway | PPT2 | 14 | P23443, Q9Y243, P31751, P10415, P36507, P05771, Q02750, P40763, Q15118, P29474, Q92793, P08887, Q09472, P06730 |
| FoxO signaling pathway | PPT2 | 19 | Q13131, P31749, Q9Y243, P31751, Q8WWL7, O95067, O00141, O15530, P36507, Q02750, P84022, P14635, P62993, P24385, P40763, Q99873, Q8WTS6, Q92793, Q09472 |
| MicroRNAs in cancer | PPT2 | 19 | O75582, O96013, P04637, O96020, P10415, P36507, P05771, Q02750, P62993, P24864, P24385, O94925, P40763, Q9Y6K1, P26358, Q92793, P42574, Q09472, Q53EL6 |
| Neuroactive ligand-receptor interaction | PPT1 | 34 | P07477, Q9H228, Q99500, P21453, O95977, O95136, P43681, P35368, P35348, P34972, P34969, P28566, P28222, P28221, P21918, P08908, P47869, Q16445, P18505, P18507, P47870, O00591, P34903, Q8N1C3, P78334, Q99928, P31644, O14764, P48169, P14867, P28472, Q9UN88, P35030, P07478 |
| Apoptosis | PPT2 | 14 | Q9Y243, P31751, P04637, Q07817, P10415, O15530, P36507, Q02750, Q13490, P09874, Q9Y6F1, Q9UGN5, P42574, Q9Y6K9 |
| Kaposi sarcoma-associated herpesvirus infection | PPT2 | 14 | Q9Y243, P31751, P04637, Q8NEB9, P36507, Q02750, P24385, P40763, Q92793, P42574, Q9Y6K9, P40189, P06400, Q09472 |
| Human immunodeficiency virus 1 infection | PPT2 | 18 | O14757, P31749, O96013, Q13153, P23443, Q9Y243, P31751, Q8WWL7, O95067, Q07817, P10415, P36507, P05771, Q02750, P14635, P42574, Q9Y6K9, Q9HC16 |
| Human cytomegalovirus infection | PPT2 | 19 | P23443, Q9Y243, P31751, P17612, O75116, P04637, P22612, P36507, P05771, Q02750, P62993, P24385, P40763, P13500, P63092, P42574, Q9Y6K9, P08887, P06400 |
| Cellular senescence | PPT2 | 20 | O14757, P31749, Q9H2X6, O96017, Q9Y243, P31751, P04637, Q8WWL7, O95067, O96020, P36507, P20248, Q02750, P84022, P14635, P24864, P24385, P78396, Q09028, P06400 |
| p53 signaling pathway | PPT2 | 11 | O96017, P04637, O95067, O96020, Q07817, P10415, P14635, P24864, P24385, P31947, P42574 |
| Pancreatic cancer | PPT2 | 11 | P23443, Q9Y243, P31751, P04637, Q07817, Q02750, P84022, P24385, P40763, Q9Y6K9, P06400 |
| Colorectal cancer | PPT2 | 11 | P23443, Q9Y243, P31751, P04637, P10415, P36507, Q02750, P84022, P62993, P24385, P42574 |
| Hepatocellular carcinoma | PPT2 | 12 | P23443, Q9Y243, P31751, P04637, Q07817, P36507, P05771, Q02750, P84022, P62993, P24385, P06400 |
| Chronic myeloid leukemia | PPT2 | 12 | Q9Y243, P31751, P04637, Q07817, P36507, P11274, Q02750, P84022, P62993, P24385, Q9Y6K9, P06400 |
| EGFR tyrosine kinase inhibitor resistance | PPT2 | 12 | P23443, Q9Y243, P31751, Q07817, P10415, P36507, P05771, Q02750, P62993, P40763, P08887, P06730 |
| Wnt signaling pathway | PPT2 | 13 | P17612, O75116, P04637, P22612, P68400, P49768, P67870, P05771, P84022, P24385, Q92793, Q9H237, Q09472 |
| Neurotrophin signaling pathway | PPT2 | 13 | O75582, Q9Y243, P31751, Q05655, P04637, P49810, P49768, P10415, P51812, O15530, P36507, Q02750, P62993 |
| Growth hormone synthesis, secretion and action | PPT2 | 13 | Q9Y243, P31751, P17612, P22612, Q13936, P36507, P05771, Q02750, P62993, P40763, P63092, Q92793, Q09472 |
| JAK-STAT signaling pathway | PPT2 | 13 | Q9Y243, P31751, Q07817, P10415, P62993, P24385, P40225, P40763, Q92793, P40189, P42702, P08887, Q09472 |
| mTOR signaling pathway | PPT2 | 14 | Q13131, P31749, P23443, Q9Y243, P31751, O00141, P51812, O15530, P36507, P05771, Q02750, P62993, Q96B36, P06730 |
| Gastric cancer | PPT1 | 3 | P28702, P10826, Q15465 |
| Gastric cancer | PPT2 | 14 | P31749, P23443, Q9Y243, P31751, P04637, O96020, P10415, P36507, Q02750, P84022, P62993, P24864, P24385, P06400 |
| Nicotine addiction | PPT1 | 17 | P43681, P47869, Q16445, P18505, P18507, P47870, O00591, P34903, Q8N1C3, P78334, Q99928, P31644, O14764, P48169, P14867, P28472, Q9UN88 |
| Hepatitis B | PPT2 | 20 | Q9Y243, P31751, P04637, O96020, P10415, P36507, P05771, P20248, Q02750, P84022, P62993, P24864, P78396, P40763, O00571, Q92793, P42574, Q9Y6K9, P06400, Q09472 |
| Serotonergic synapse | PPT1 | 10 | P46098, P34969, P28566, P28222, P28221, P08908, P18505, P47870, P28472, P51589 |
| Serotonergic synapse | PPT2 | 12 | P17612, P22612, P18054, Q13936, O15296, P05771, Q02750, P09917, P63092, P42574, Q00975, P05067 |
| Morphine addiction | PPT1 | 19 | Q16445, P18505, P18507, P47870, O00591, P34903, Q8N1C3, P78334, Q99928, P31644, O14764, P48169, P14867, P28472, Q9UN88, Q08499, P27815, Q07343, Q08493 |
| Morphine addiction | PPT2 | 5 | P17612, P22612, P05771, P63092, Q00975 |

**Table 4. Compound list**

| **Drug** | **Cluster** | **Mechanism/Targets** | **Concentration (nM)** | **Solvent** | **Supplier Ref** | **Supplier** |
| --- | --- | --- | --- | --- | --- | --- |
| LY3009120 | 1 | pan-RAF inhibitor | 1-10000 | DMSO | HY-12558 | Medchem Express |
| Ruxolitinib | 1 | JAK1&2 inhibitor | 1-10000 | DMSO | CT-INCB-2 | ChemieTek |
| Birabresib | 1 | BET family inhibitor | 1-10000 | DMSO | S7360-2 | Selleck |
| Plicamycin | 1 | RNA synthesis inhibitor | 1-10000 | DMSO | sc-200909-7 | Santa Cruz Biotechnology |
| Teniposide | 2 | Topoisomerase II inhibitor | 1-10000 | DMSO | HY-13761 | Medchem Express |
| Silmitasertib | 2 | CSNK2A1 inhibitor | 1-10000 | DMSO | S2248 | Selleck |
| Ulixertinib | 2 | ERK inhibitor | 1-10000 | DMSO | CT-VRT752 | ChemieTek |
| Sapanisertib | 2 | mTOR1/2 Inhibitor | 0.1-1000 | DMSO | CT-INK128 | ChemieTek |

**Table 5. Patient characteristics**

| **Sample_ID** | **Diagnosis** | **Disease Stage** | **FAB** | **genetic Characteristics** | **age** | **Malignant Cell Percentage** |
| --- | --- | --- | --- | --- | --- | --- |
| AML_1 | C92.0 Acute myeloid leukaemia [AML] | Refractory | M5 | ["+8","+11"] | 67 | 59 |
| AML_2 | C92.0 Acute myeloid leukaemia [AML] | Relapse |  | ["Chromosomal abnormalities not checked"] | 75 | 49 |
| AML_3 | C92.0 Acute myeloid leukaemia [AML] | Diagnosis | M5 | ["No chromosomal abnormalities detected"] | 34 | 85 |
| AML_4 | C92.0 Acute myeloid leukaemia [AML] | Diagnosis | M2 | ["No chromosomal abnormalities detected"] | 44 | 55 |
| AML_5 | C92.0 Acute myeloid leukaemia [AML] | Relapse | M5 | ["No chromosomal abnormalities detected"] |  | 75 |
| AML_6 | C92.0 Acute myeloid leukaemia [AML] | Relapse | M0,FAB M1 | Not available |  | 83 |
| AML_7 | C92.0 Acute myeloid leukaemia [AML] | Relapse |  | ["inv(11)","add(14q)","inc[cp9]/46"] | 55 | 90 |
| AML_8 | C92.0 Acute myeloid leukaemia [AML] | Diagnosis | M2 | ["No chromosomal abnormalities detected"] | 75 | 90 |
| AML_9 | C92.0 Acute myeloid leukaemia [AML] | Diagnosis |  | ["No chromosomal abnormalities detected"] | 72 | 77 |
| AML_10 | C92.4 Acute promyelocytic leukaemia [PML] | Diagnosis | M3 | ["t(15;17)(q22;q12); PML-RARA*"] | 56 | 80 |
| AML_11 | C92.4 Acute promyelocytic leukaemia [PML] | Diagnosis | M3 | ["t(15;17)(q22;q12); PML-RARA*"] | 60 | 60 |
| AML_12 | C92.0 Acute myeloid leukaemia [AML] | Relapse |  | ["t(xx;11)(xx;q23) MLL-fusions"] | 7 | 100 |
| AML_13 | C92.0 Acute myeloid leukaemia [AML] | Diagnosis |  | ["t(8;21)(q22;q22); RUNX1-RUNX1T1","abn(11)"] | 72 | 86 |
| AML_14 | C92.0 Acute myeloid leukaemia [AML] | Diagnosis |  | ["No chromosomal abnormalities detected"] | 58 | 82 |
| AML_15 | C92.0 Acute myeloid leukaemia [AML] | Diagnosis |  | Not available | 64 | 53 |
| AML_16 | C92.0 Acute myeloid leukaemia [AML] | Diagnosis |  | Not available | 52 | 81 |
| Healthy_1 | Healthy |  |  |  |  |  |
| Healthy_2 | Healthy |  |  |  |  |  |
| Healthy_3 | Healthy |  |  |  |  |  |
| Healthy_4 | Healthy |  |  |  |  |  |
| Healthy_5 | Healthy |  |  |  | 66 |  |

**Table 6. Protein target analysis of five investigated drugs.**

| **Drug1** | **Drug2** | **Target D1** | **Target D2** | **Overlap** | **P-value** |
| --- | --- | --- | --- | --- | --- |
| Sapanisertib | LY3009120 | 49 | 24 | 2 | 0.98 |
| Ulixertinib | Ruxolitinib | 4 | 150 | 1 | 0.98 |
| Silmitasertib | Ruxolitinib | 52 | 150 | 26 | 1 |
| Silmitasertib | LY3009120 | 52 | 24 | 0 | 1 |
| Ulixertinib | LY3009120 | 4 | 24 | 0 | 1 |

**Table 7.** **Antibodies used in flow cytometry.**

| **Biomarker** | **Fluorophore** | **Clone** | **iQue3 channel** | **Dilution** | **Manufacturer** | **Ref. No** |
| --- | --- | --- | --- | --- | --- | --- |
| CD38 | BV421 | HIT2 | VL1 | 1:800 | BD Biosciences | 562444 |
| CD11b | BV605 | ICRF44 | VL4 | 1:267 | BD Biosciences | 562721 |
| CD15 | BV786 | W6D3 | VL6 | 1:800 | BD Biosciences | 741013 |
| CD34 | PE | 563 | BL2 | 1:160 | BD Biosciences | 550761 |
| CD45 | V500 | HI30 | VL2 | 1:267 | BD Biosciences | 560777 |
| CD14 | APC | M5E2 | RL1 | 1:62 | BD Biosciences | 555399 |
| CD117 | PE/Cy7 | 104D2 | BL5 | 1:800 | Biolegend | 313212 |
| DRAQ7 | N/A | N/A | RL2 | 1:800 | BD Biosciences | 564904 |
| Annexin V | FITC | N/A | BL1 | 1:200 | BD Biosciences | 556419 |

**Table 8. FC results in different populations**

| **Drug Combinations** | **Sample** | **Live Ratio** | **Blast Ratio** | **Response** | **CD117**  **Ratio** | **Response** | **CD34**  **Ratio** | **Response** | **CD14**  **Ratio** | **Response** | **CD15**  **Ratio** | **Response** | **Imphocyte Ratio** | **Response** | **CD34+CD38-**  **Ratio** | **Response** | **CD34+CD38+**  **Ratio** | **Response** | **CD11b**  **Ratio** | **Response** |
| --- | --- | --- | --- | --- | --- | --- | --- | --- | --- | --- | --- | --- | --- | --- | --- | --- | --- | --- | --- | --- |

**LY3009120 &**

**Sapanisertib**

**LY3009120 &**

**Silmitasertib**

**LY3009120 &**

**Ulixertinib**

**Ruxolitinib & Silmitasertib**

**Ruxolitinib & Ulixertinib**

AML_3

Average

0.84

0.65

0.76

0.86

32.57

47.01

0.48

0.25

33.22

36.57

0.59

0.23

33.84

48.61

0.25

0.09

21.19

43.91

0.32

0.20

28.75

52.85

0.03

0.03

3.45

21.37

0.55

0.18

22.96

21.52

0.25

0.12

52.35

58.55

0.18

0.07

48.06

56.54

**Total average**

0.65

0.69

35.70

0.26

30.96

0.30

34.88

0.17

56.47

0.24

54.75

0.06

13.75

0.25

35.11

0.09

55.24

0.11

55.60

AML_4 0.83 0.94 42.7 0.24 0 0.06 30.97 0 33.19 0.11 47.08 0.03 1.75 0.00 0.00 0.05 41.44 0.02 38.84

AML_1 0.77 0.16 53.37 0.05 39.7 0.03 42.75 0.54 74.98 0.35 70.83 0.04 5.65 0.03 69.47 0.07 80.53 0.29 77.14

AML_2 0.56 0.76 23.53 0.49 14.44 0.75 26.7 0 89.93 0.23 77.18 0.19 9.44 0.67 25.99 0.04 70.77 0.02 62.87

Average 0.72 0.56 39.49 0.34 29.47 0.46 37.61 0.26 79.50 0.30 71.78 0.09 7.77 0.42 46.72 0.12 70.65 0.16 71.34

AML_3 0.84 0.76 41.56 0.48 34.26 0.59 43.38 0.25 73.6 0.32 67.32 0.03 8.21 0.55 44.70 0.25 60.64 0.18 74.00

AML_2 0.56 0.76 13.48 0.49 10.01 0.75 14.08 0 81.54 0.23 61.66 0.19 4.94 0.67 13.05 0.04 45.12 0.02 41.56

AML_4 0.83 0.94 27.09 0.24 27.71 0.06 5.1 0 28.26 0.11 22.23 0.03 3.45 0.00 20.70 0.05 6.99 0.02 25.11

AML_1 0.77 0.16 21.41 0.05 18.12 0.03 17.2 0.54 45.28 0.35 41.73 0.04 5.42 0.03 51.78 0.07 52.72 0.29 48.89

Average 0.72 0.62 20.66 0.26 18.61 0.28 12.13 0.18 51.69 0.23 41.87 0.09 4.60 0.24 28.51 0.05 34.94 0.11 38.52

AML_2 0.56 0.76 11.64 0.49 6.33 0.75 12.93 0 77.35 0.23 64.3 0.19 3 0.67 12.10 0.04 52.39 0.02 45.32

AML_1 0.77 0.16 24.86 0.05 33.82 0.03 31.8 0.54 52.97 0.35 48.24 0.04 2.79 0.03 53.36 0.07 56.09 0.29 52.93

AML_5 0.27 0.89 46.47 0.02 68.86 0.04 51.27 0.03 52.94 0.16 55.41 0.02 44.94 0.00 61.06 0.04 50.26 0.02 56.25

Average 0.53 0.60 27.66 0.19 36.34 0.27 32.00 0.19 61.09 0.25 55.98 0.08 16.91 0.23 42.17 0.05 52.91 0.11 51.50

AML_5 0.27 0.89 66.46 0.02 75.83 0.04 80.45 0.03 74.44 0.16 82.02 0.02 53.01 0.00 78.37 0.04 80.50 0.02 77.02

AML_4 0.83 0.94 41.86 0.24 0.85 0.06 32.66 0 31.43 0.11 38.44 0.03 2.26 0.00 2.52 0.05 41.21 0.02 44.50

AML_3 0.84 0.76 31.25 0.48 29.04 0.59 29.29 0.25 23.17 0.32 31.89 0.03 4.76 0.55 18.88 0.25 58.15 0.18 59.55

Average 0.65 0.86 46.52 0.25 35.24 0.23 47.47 0.09 43.01 0.20 50.78 0.03 20.01 0.18 33.25 0.12 59.95 0.07 60.36

AML_5 0.27 0.89 65.75 0.02 76.48 0.04 81.02 0.03 77.35 0.16 82.71 0.02 58.9 0.00 41.59 0.04 81.87 0.02 82.72
